# Psychobiotic interventions for anxiety in young people: a systematic review and meta-analysis, with youth consultation

**DOI:** 10.1038/s41398-021-01422-7

**Published:** 2021-06-16

**Authors:** Kathrin Cohen Kadosh, Melissa Basso, Paul Knytl, Nicola Johnstone, Jennifer Y. F. Lau, Glenn R. Gibson

**Affiliations:** 1 School of Psychology, Faculty of Health and Medical Sciences, University of Surrey, Guildford, UK; 2 Department of Psychology, Institute of Psychiatry Psychology & Neuroscience, King’s College London, London, UK; 3 Department of Food and Nutritional Sciences, The University of Reading, Reading, UK

**Keywords:** Physiology, Human behaviour

## Abstract

The human gut microbiome influence on brain function and mental health is an emerging area of intensive research. Animal and human research indicates adolescence as a sensitive period when the gut-brain axis is fine-tuned, where dietary interventions to change the microbiome may have long-lasting consequences for mental health. This study reports a systematic review and meta-analysis of microbiota-targeted (psychobiotics) interventions on anxiety in youth, with discussion of a consultation on the acceptability of psychobiotic interventions for mental health management amongst youth with lived experience. Six databases were searched for controlled trials in human samples (age range: 10–24 years) seeking to reduce anxiety. Post intervention outcomes were extracted as standard mean differences (SMDs) and pooled based on a random-effects model. 5416 studies were identified: 14 eligible for systematic review and 10 eligible for meta-analysis (total of 324 experimental and 293 control subjects). The meta-analysis found heterogeneity *I*^2^ was 12% and the pooled SMD was −0.03 (95% CI: −0.21, 0.14), indicating an absence of effect. One study presented with low bias risk, 5 with high, and 4 with uncertain risk. Accounting for risk, sensitivities analysis revealed a SMD of −0.16 (95% CI: −0.38, 0.07), indicative of minimal efficacy of psychobiotics for anxiety treatment in humans. There is currently limited evidence for use of psychobiotics to treat anxiety in youth. However, future progress will require a multidisciplinary research approach, which gives priority to specifying mechanisms in the human models, providing causal understanding, and addressing the wider context, and would be welcomed by anxious youths.

## Introduction

**Fig. 1:**
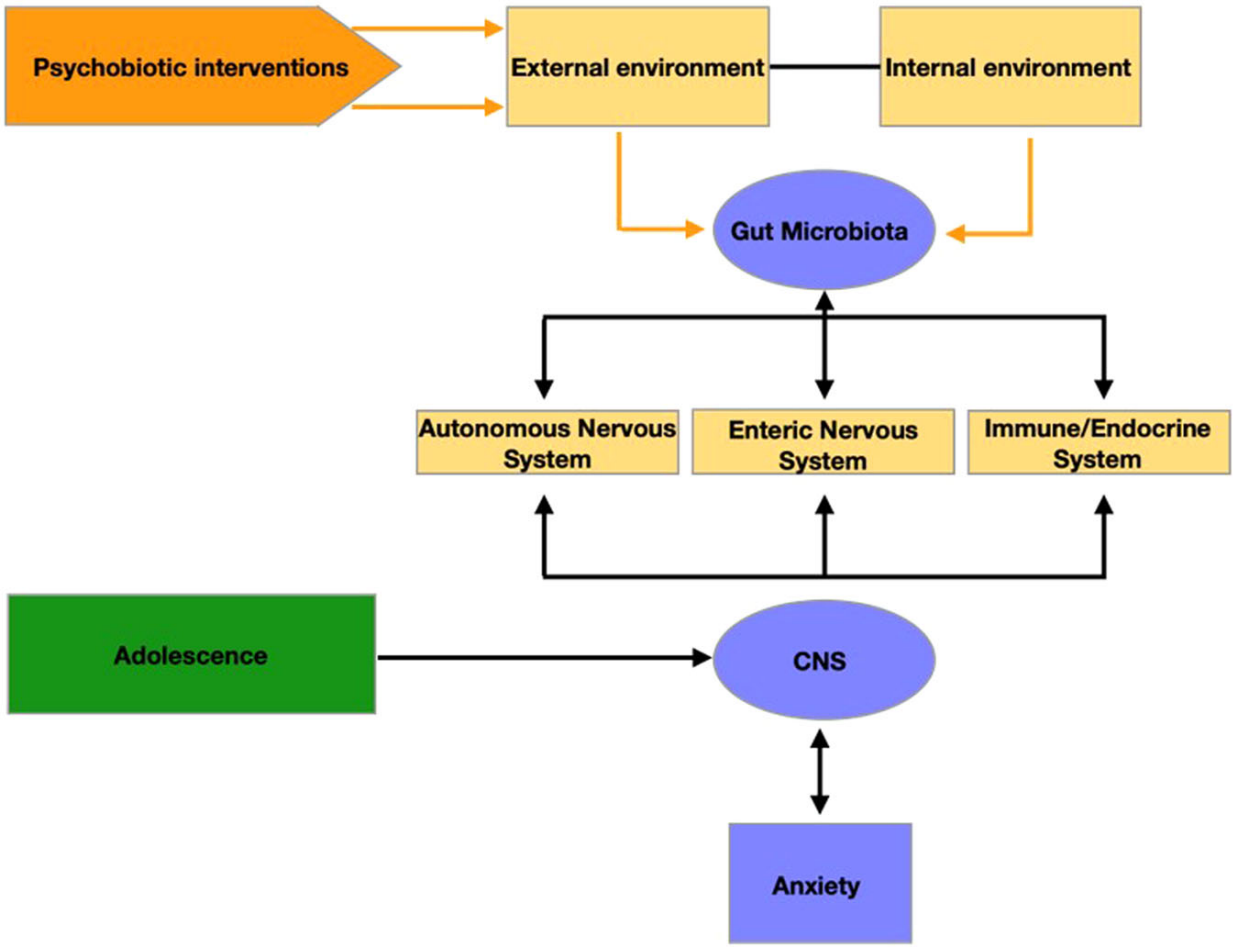
Proposed intervention pathway for the active ingredient. Adolescence is a time period of ongoing neuro-cognitive development, allowing brain structures and circuities to flexibly adapt- or maladapt to the environment. In this context, gut microbiota might play be a causal role as a mediator between the environment and the CNS via multiple pathways. As easily manipulated throughout diet, it could be a promising and cheap therapy target in the redirection of neurodevelopmental trajectories and improving the mental health outcome for the individual.

Altering the gut microbiota with nutritional therapeutics such as psychobiotics (i.e. active compounds such as probiotics and prebiotics) shows promise in treating mental health problems such as depression and anxiety. Recent research on psychobiotics as active ingredients in host physiology shows influence on the nervous system, consequentially shaping psychological processes, behaviour and ultimately exerting health benefits in psychiatric conditions in preclinical animal research^1–4^ and in humans^5–8^. Animal research has also shown that variations in gut bacteria composition may lead to the psychological abnormalities that characterize anxiety^3,9,10^ (see also refs. ^11,12^ for a review). Therefore, manipulation of the gut microbiome via psychobiotics may present a promising new avenue for treatment and prevention of anxiety in young people^11,12^ (see Fig. 1 for a proposed pathway).

One class of psychobiotics are known as probiotics—live gut bacteria capable of releasing neuroactive substances^13^, depending on the bacterial strain. For example, the *Bifidobacterium* family is linked to GABA expression in the brain, whereas the *Enterococcus* and *Streptococcus* families are shown to produce serotonin, and *Lactobacillus* are linked to GABA and acetylcholine^12^. Animal research has consistently outlined the psychotropic effects of probiotics. For example, Barrett et al.^14^ showed that *Lactobacillus (L). brevis* and *Bifidobacterium (B.) dentium* increased GABA concentrations in-vitro, a finding which was replicated in an in-vivo model which showed that a *L. brevis* ingested strain regulated emotional behavioural and central GABA receptor expression in a mouse model^1^. In this study, mice fed *L. rhamnosus* exhibited reduced GABA mRNA expression in the amygdala, along with lower levels of stress-induced corticosterone and anxiety- and depression-related behaviour. Another study replicated this finding^15^ and showed that increases in GABA metabolites in the brain were evident after about 4 weeks, a lag comparable with other pharmaceutical interventions, such as serotonin-reuptake inhibitors^16^. Notably, the same study pointed out that re-colonization of gut bacteria in adolescent germ-free (GF) mice (i.e. mice without any microorganisms living in or on them) was not sufficient to reverse anxiety-like behaviour, suggesting that early deficits in gut microbiota may not be reversible.

Along with probiotics, some prebiotics are classified as psychobiotics. Prebiotics are specific non-digestible food ingredients (including non-digestible oligosaccharides) which selectively feed intrinsic beneficial bacteria, consequentially stimulating their growth and activity with notable effects on brain development and function^12,17^. To date, fructooligosaccharides (FOS) and galactooligosaccharides (GOS) have been the most studied prebiotics, showing promising effects in animal and human trials^2,18^. In the context of cognitive function, Tarr et al. ^18^ have shown that milk oligosaccharides administration can prevent stress-induced dysbiosis and anxiety-like behaviour in mice. Likewise, Burokas et al. ^2^ have reported chronic combined FOS and GOS supplementation in mice to have anxiolytic and antidepressant effects, as well as to reduce corticosterone stress response. In addition, prebiotics have been shown to modulate hippocampal and hypothalamus gene expression, and lead to SCFAs concentration changes which positively correlate with the behavioural effects.

Animal research has shown that adolescence is a critical window where microbiota help fine-tune the gut–brain axis^19^ and, given the link between the gut–brain axis and mental health, could be one factor for a significant increase in mental health problems during this period^20,21^. This makes a healthy gut microbiome an important and possibly time-sensitive active ingredient, as interventions during this period may have long-lasting consequences both at the gut microbiome and brain level. One study reported that rats’ ingestion of pre-gestational *Lactobacillus helveticus* resulted in offspring which then displayed lower rates of anxiety-like behaviour in adolescence^4^. In young rats who were experimentally stressed, those who received a placebo presented with abnormal pubertal timing, whilst those fed *Lactobacillus rhamnosus* and *Lactobacillus helveticus* presented normal timing^22^. In another study, adolescent mice were injected with the toxin lipopolysaccharide which caused immediate and temporary depression- and anxiety-like behaviour, but also increased stress-sensitivity in adulthood. Compared to placebo, mice who received a mixture of *Lactococcus lactis, L. cremoris, L. diacetylactis, L. acidophilus* around the time of toxin administration displayed shorter duration of immediate negative effects and also did not display as severe stress-responses in adulthood^23^. These findings support an earlier landmark study^24^ which found that adult GF mice had enhanced stress responses which could be reversed by gut colonization with *Bifidobacterium infantis*. Crucially, the earlier in the lifespan this intervention took place, the more fully a normal stress response was restored. Thus, if these promising effects translate to humans, psychobiotics present candidate ingredients which could provide a measure of protection against stress-induced anxiety in adolescents which may carry over into adulthood. Research will also need to exert caution when matching age-range from non-human participants to human participants, as sensitive periods may differ.

The aim of this review is to capture the current state of the literature on psychobiotics for anxiety and stress reduction in youths aged 10–24 years old in comparison to placebo/treatment as usual to evaluate the translational efficacy of psychobiotic use in development.

## Methods

### Protocol

A systematic review and meta-analysis were conducted to evaluate the effect of psychobiotics on anxiety and stress in human youth aged 10–24 years. Trials were selected where an active treatment (probiotics or prebiotics) and placebo and/or treatment as usual group were included. Primary outcome was anxiety symptomology improvement based on questionnaire or interview response due to active treatment, and secondary outcomes examined behavioural anxiety indices (for example, performance on the emotional Stroop task) and stress measures. Method reporting was consistent with PRISMA statement guidelines (for the PRISMA checklist see Appendix 5).

### Selection criteria

Controlled trials assessing anxiety and stress as primary or secondary outcomes with at least one active treatment group and one comparator group were included. Inclusion criteria were as follows: (1) Mean age in the range of 10–24 years old; (2) healthy and clinical samples; (3) minimally measures obtained pre- and post-intervention; (4) pro- or prebiotic administration (any form); (5) published and peer-reviewed data; (6) any date of publication. Exclusion criteria were: (1) administration of pro- and prebiotic combinations (synbiotics); (2) unpublished data; (3) duplicate data/publications.

### Search strategy, study selection and data extraction

6 databases were searched (PubMed, Embase, Cochrane, Scopus, Ovid, Web of Science) between the 30^th^ of May and 10^th^ of June 2020 using the search terms reported in Appendix 1 with no publication date restrictions. 5416 studies were identified and imported into EppiReviewer4 for duplicate identification and removal (1549). Studies were screened independently by authors MB and PK, and conflicts resolved in consultation with KCK. Qualitative data detailing author, year of publication, intervention type (pro- or prebiotic), form of psychobiotic administration (e.g. liquid, powder), active compound (e.g. bacterial strain), dose (e.g. CFU or mg), frequency (e.g. daily), duration of treatment (e.g. in days), sample size, age, gender and outcome measure were managed in excel spreadsheets; and quantitative data on outcome effects in Review Manager 5. Where data could not be inferred from publication, study authors were contacted via email.

Decisions for the exclusion of studies were based on the assessment of the included sample, administered intervention, study design and presence of a control group. (1) Samples with mean age below 10 years old and above 24 years old were excluded as highly likely to fall outside the adolescence age range which is thought to start with puberty (~10) and ends as one takes on adult social roles^25^. (2) Symbiotic interventions were excluded to avoid any confusion due to interaction effect between the two compounds. (3) Observational trials, non-control trials and non-published/ non-peer reviewed data were excluded to ensure good research quality.

### Risk of bias assessment

Studies reporting anxiety outcomes were assessed for risk of bias on the outcome, and for studies reporting on stress outcomes, risk of bias evaluated on the study. The Revised Cochrane risk-of-bias tool for randomization trials (RoB-2)^26^ was used to consider the following domains for bias; (1) random sequence generation (selection bias); (2) allocation concealment (selection bias); (3) personnel and participant blinding (performance bias); (4) outcome assessment blinding (detection bias); (5) incomplete outcome data (attrition bias); (6) selective reporting (reporting bias); (7) other sources of bias. The Excel tool of RoB2 available at Risk of bias tools—Current version of RoB 2 was used to support the assessment and the creation of the risk of bias figure. Finally, Review Manager 5 (RevMan5)^27^ was used to create a funnel plot in order to assess the presence of biases affecting the cumulative evidence.

### Statistical analysis

Data were extracted as standardized mean differences (SMDs) with 95% CI and *I*^2^ statistics used as a between-studies heterogeneity index. The pooled SMD was calculated based on a random-effects model using Review Manager 5. Sensitivity analyses were also performed removing the studies at high risk of bias from the analyses.

## Results

### Study selection

**Fig. 2:**
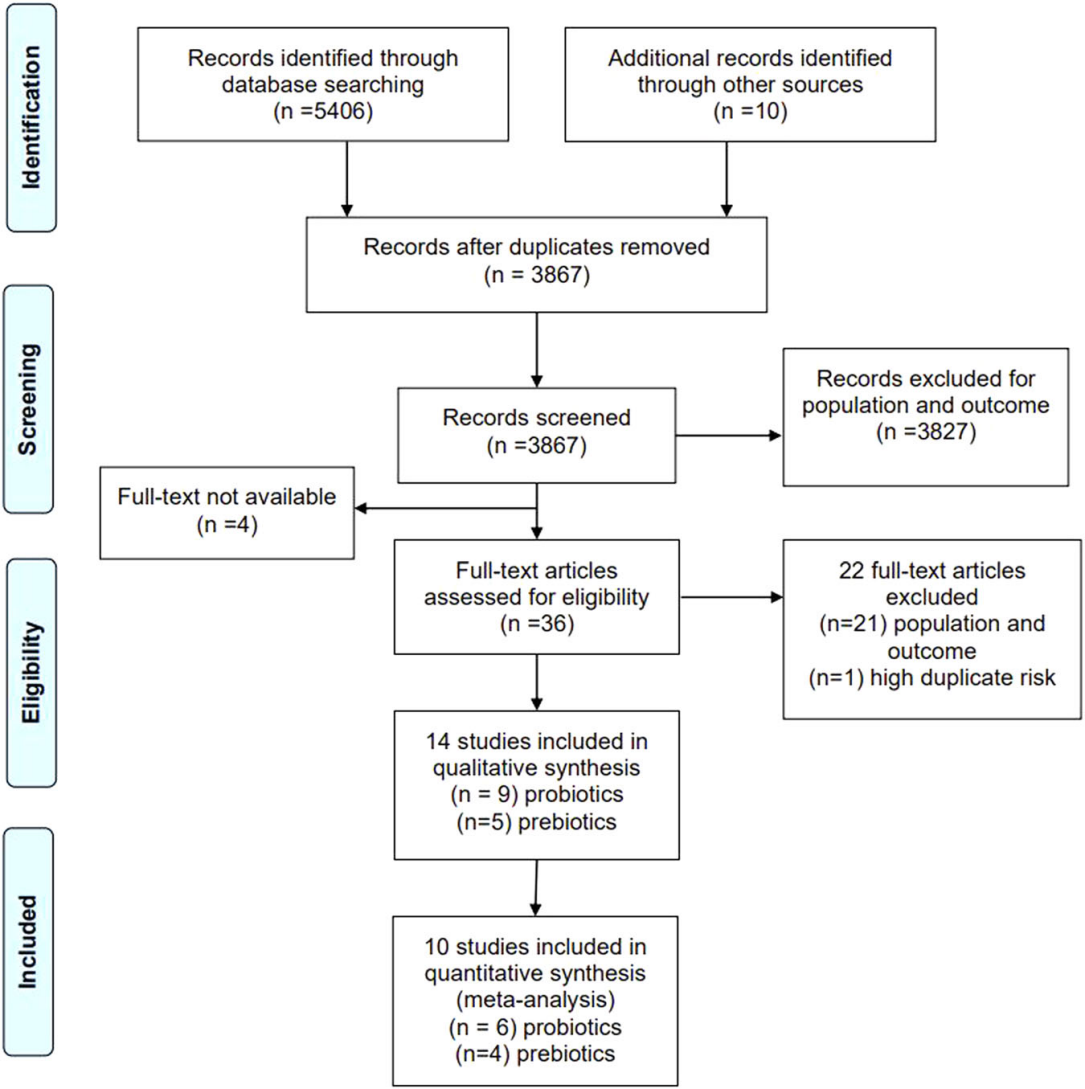
PRISMA flowchart of search results at each step of the systematic review. This illustarates the number of studies considered for inclusion and exclusion throughout the study.

3867 abstracts and titles were screened independently (by authors MB and PK). 3827 were excluded for ineligible samples and outcomes and 4 for text unavailability. 36 full-text studies remained for eligibility assessment; 21 were excluded for ineligible samples and outcomes while 1 was excluded for using the same participant sample in two included studies^28,29^. The final output for the systematic review was 14 studies, 9 using probiotic interventions, and 5 using prebiotic interventions that are presented in Table 1. 10 studies were included in the meta-analyses (Fig. 2).

**Table 1:** Characteristics of the studies included in the systematic review.

Study	Intervention Type	Delivery Method	Active Compound	Dose	Frequency (dose/day)	Duration (days)	Active/Control	Mean Age (SE)	Sex (M/F)	Anxiety Measure*	Effect	Stress Measure*	Effect2	Participants
Andersson et al.^35^	probiotic	capsule	*Lactobacillus plantarum 299v*	10 x 10^9^ CFU	1	21	21/20	range 18-30	13/28	-	-	salivary cortisol	↓	students e.s.
												salivary immunoglobulin A	ns	
Culpepper et al.^5^	probiotic	capsule	*Lactobacillus helveticus* R0052	3 × 10^9^ CFU	1	42	145	19.9 (0.1)	209/372	-	-	self-reported stress	↓ for *B. Bifidum* only, only in sleep deprived students	students e.s.
			*Bifidobacterium longum ssp.* infantis R0033				147							
			*Bifidobacterium bifidum* R0071				142							
			placebo				147							
Hughes et al. (2011)	prebiotic	sachet	galactooligosaccharide	0, 2.5, 5 g	1	56	279/140	19.9 (0.1)	207/212	-	-	self-reported stress	ns	students e.s.
Karbownik et al.^34^	probiotic	capsule	*Saccharomyces boulardii*	5 x 10^9^ CFU	1	30	31/29	22.6	37/55	STAI state	ns	salivary cortisol	ns	students e.s.
												salivary metanephrine	ns	
												pulse rate	↑	
Kato-Kataoka et al.^28^	probiotic	liquid	*Lactobacillus casei* Shirota	100 x 10^9^ CFU	1	56	23/24	22.8 (0.4)	25/22	STAI state	ns	visual analogue stress scale	↓	students e.s.
												salivary cortisol	↓	
												salivary alpha-amylase	ns	
Kato-Kataoka et al.^29^	probiotic	liquid	*Lactobacillus casei* Shirota	100 x 10^9^ CFU	1	56	24/23	22.9 (0.4)	26/21	STAI state	ns	salivary cortisol	ns	students e.s.
										HADS	-	salivary immunoglobulin A	ns	
Kitaoka et al.^39^	prebiotic	capsule	Fermented Ginseng	205 mg	9	8	8/8	20.7 (0.4)	16/0	STAI total	-	salivary cortisol	ns	healthy subjects
										POMS	ns	salivary immunoglobulin A	ns	
												urinary 8-hydroxydeoxyguanosine	ns	
Kiecolt-Glaser et al.^38^	prebiotic	capsule	omega-3 polyunsaturated fatty acid	2.5 g	1	84	34/34	23.7 (1.9)	38/30	BAI	↓	-	-	students e.s.
Liu et al. (2019)	probiotic	capsule	*Lactobacillus plantarum* PS128	30 x 10^9^ CFU	ns	30	36/35	10.0	71/0	CBCL	ns	-	-	ASD children
Manos et al.^36^	prebiotic	capsule	omega-3-PUFA	782 mg	4	84	10/8	14.7	0/18	BAIT	↑	-	-	anorexic girls
Marcos et al.^33^	probiotic	liquid	*Lactobacillus delbrueckii bulgaricus*	1 x 10^9^ CFU	2	21	73/63	18-23	40/96	STAI state	ns	serum cortisol	ns	students e.s.
			*Streptococcus salivarius thermophilus*	10 x 10^9^ CFU						STAI trait	ns			
			*Lactobacillus casei* DN114001	10 x 10^9^ CFU										
Moller et al. (2017)	probiotic	capsule	*Bifidobacterium breve*	112.5 x 10^9^ CFU (total)	1	14	57/48	20.2	36/69	-	-	PASAT	ns	healthy subjects, stress task
			*B.longum*									Blood pressure	ns	
			*B. infantis*											
			*Lactobacillus acidophilus*											
			*L. plantarum*											
			*L. paracasei*											
			*L. bulgaricus*											
			*Streptococcus thermophilus*											
Schmidt et al. (2014)	prebiotic	powder	fructooligosaccharides (FOS)	5.5 g	1	21	15	23.7	22/23	STAI state	ns	PSS-10	ns	healthy subjects
			galactooligosaccharide (B-GOS)				15			Attentional dot-probe	↓GOS only, unmasked	salivary cortisol	↓ GOS only	
			placebo				15			Facial expression recognition	ns			
										Emotional word recognition and recall	ns			
Tran et al.^30^	probiotic	-	18 species	50 x 10^9^ CFU	1	28	14	20.6	20/66	BAI	ns BAI total ↑ BAI-Panic, 50 x 10^9 CFU only	-	-	healthy students
			10 species	50 x 10^9^ CFU			13			ACQ-R	-			
			18 species	15 x 10^9^ CFU			15			PSWQ	↓50 x 10^^9^ CFU only			
			10 species	10 x 10^9^ CFU			15							
			placebo				11							

↓: improvement vs placebo; ↑: deminishment vs placebo; *: effect size estimated or calculated from reported data; ns: no significant effect; -: not reported or not applicable; ACQ-R: Anxiety control questionnaire-revised; BAI: Beck Anxiety Inventory; CBCL: Child Behaviour Checklist (Anxiety); CFU: colony forming units; HADS: Hospital Anxiety and Depression Scale; PASAT: Paced Auditory Serial Addition Test; PSS: Percieved Stress Scale-10; PSWQ: Penn state worry questionnaire; STAI: State-Trait Anxiety Inventory; e.s.: under examination stress.

* all the time point 2 are referred to POST-TREATMENT measurement

## Probiotic interventions

### Study characteristics

Probiotic studies used many different species either singularly or in combination. Of the 9 probiotic studies, 4 used multiple species of probiotics in the same treatment group and one used up to 18 different species in a group^30,31^. Across the review, species used included *L. plantarum, helveticus, rhamnosus, casei, casei* Shirota*, paracasei, plantarum, bulgaricus, delbrueckii bulgaricus, acidophilus; B. longum, bifidum, breve, infantis; Saccharomyces boulardii; Streptococcus salivarius thermophilus;* and *Streptococcus thermophilus*. Dose size and frequency and duration of intervention also varied widely. While often in the order of tens of billions of colony-forming units (CFU), doses ranged from 10^9^ CFU to 10^11^ CFU, administered once to twice a day for a duration of 14 to 56 days.

### Heterogeneity

There was a large degree of outcome heterogeneity. When anxiety was measured in the studies, it was with a number of validated instruments such as the Beck Anxiety Inventory (BAI), State-Trait Anxiety Inventory (STAI), Hospital Anxiety and Depression Scale (HADS), or Penn State Worry Questionnaire (PSWQ). Moreover, a variety of stress measures were used, including salivary cortisol, immunoglobulin A, metanephrine, alpha-amylase; self-reported stress; blood pressure; pulse rate; serum cortisol; urinary 8-hydroxydeoxyguanosine; and performance on a behavioural task. Taken together, this heterogeneity is likely due to the novelty of the field; however, it makes direct comparison between studies more challenging. Future studies should focus on systematic replications of the findings reported in these studies and, where applicable, confirmation of effective and tolerable doses needs to be assessed.

### Outcomes

Perhaps in part because of heterogeneity in methods employed, the review revealed mixed results for the reduction of stress using probiotics in young people. Of 6 probiotic studies which measured anxiety, 5 did not find any significant effect. The remaining study ^31^, showed an improvement of worrying symptoms proxied by the PSWQ, although in only subjects administered with a high daily dose (50 x 10^9^ CFU)^30^. By far the most common study design for both pre- and probiotics involved following a cohort of selected and unselected university students before, during, and after final exams^5,28,29,32–35^. These studies often only used stress or anxiety as a secondary outcome, focussing on stress-related GI problems or immune performance, mostly with salivary cortisol as the main outcome. There was some evidence (based on the statistical analysis) to support *L. casei Shirota* having a reducing effect on salivary cortisol and self-reported stress in students approaching final exams^29,32^. A further study from another group reported reduced stress in sleep-deprived students after *B. bifidum* administration during exam periods^5^. About 50% of studies which measured stress in some way did not find significant effects of their probiotic intervention on stress. One study^34^ even reported increased pulse rate in the probiotic group (*Saccharomyces boulardii*) which could be interpreted as increased physiological stress, although there was no change in self-reported anxiety.

Taken together, the literature revealed that probiotics used had mixed results for reducing stress in youth. This makes sense given the large variety of strains available and tested. Furthermore, the literature currently does not support probiotic use in reducing anxiety, and two studies reported adverse effects, e.g. increased BAI^31^ scores and increased pulse rate^34^.

## Prebiotic interventions

### Study characteristics

Potentially more promising, but less investigated are prebiotic interventions, where 5 eligible studies were identified. A number of different prebiotic compounds were used, galactooligosaccharide, fructooligosaccharide, omega-3 polyunsaturated fatty acid, and fermented ginseng. Doses of prebiotics were more comparable, ranging from 1.8 g/day to 5.5 g/day administered for 8 to 56 days; however, study duration was also variable from 8 to 84 days. Dose per day was typically once, with one study requiring 4 doses per day^36^.

### Outcomes

Of the five studies that met our inclusion criteria, no significant effects were found on participant stress. A study of girls with *anorexia nervosa*^36,37^ reported no significant effect of omega-3 polyunsaturated fatty acid (PUFA) on anxiety as measured by the Beck Anxiety Inventory-trait (BAIT)—however this may have been due to lack of statistical power (*n* = 18). Note that while prebiotic status of PUFA is not yet universally accepted, new evidence supports the inclusion here^37^. A larger study also used PUFA as the active ingredient (*n* = 68)^38^; but reported reduced BAI in otherwise healthy university students. Another small study (*n* = 16) investigating the effects of fermented ginseng^39^ reported reduced total STAI score in healthy university students in the intervention group only. Finally, one study using GOS^6^ reported both reduced attention to negatively valenced stimuli (hypervigilance to negative stimuli is associated with anxiety and depression) and decreased salivary cortisol (although STAI was unaffected). Furthermore, unpublished work by our lab replicates this vigilance reducing effect of GOS in young females^27^, along with a reduction in self-reported trait anxiety levels. Interestingly, in this study^27^, a reduction in self-reported anxiety levels (STAIT) was only found in participants with high trait anxiety, which suggests that prebiotic interventions may be most effective in cases where there is already some evidence of difficulties with emotion regulation.

## Meta-analysis of anxiety outcomes

**Fig. 3:**
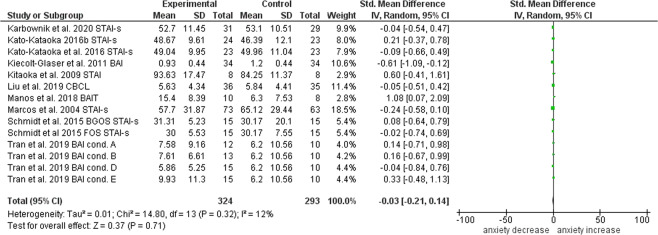
Forest plot of the studies investigating the effect of psychobiotics on anxiety measures.

**Fig. 4:**
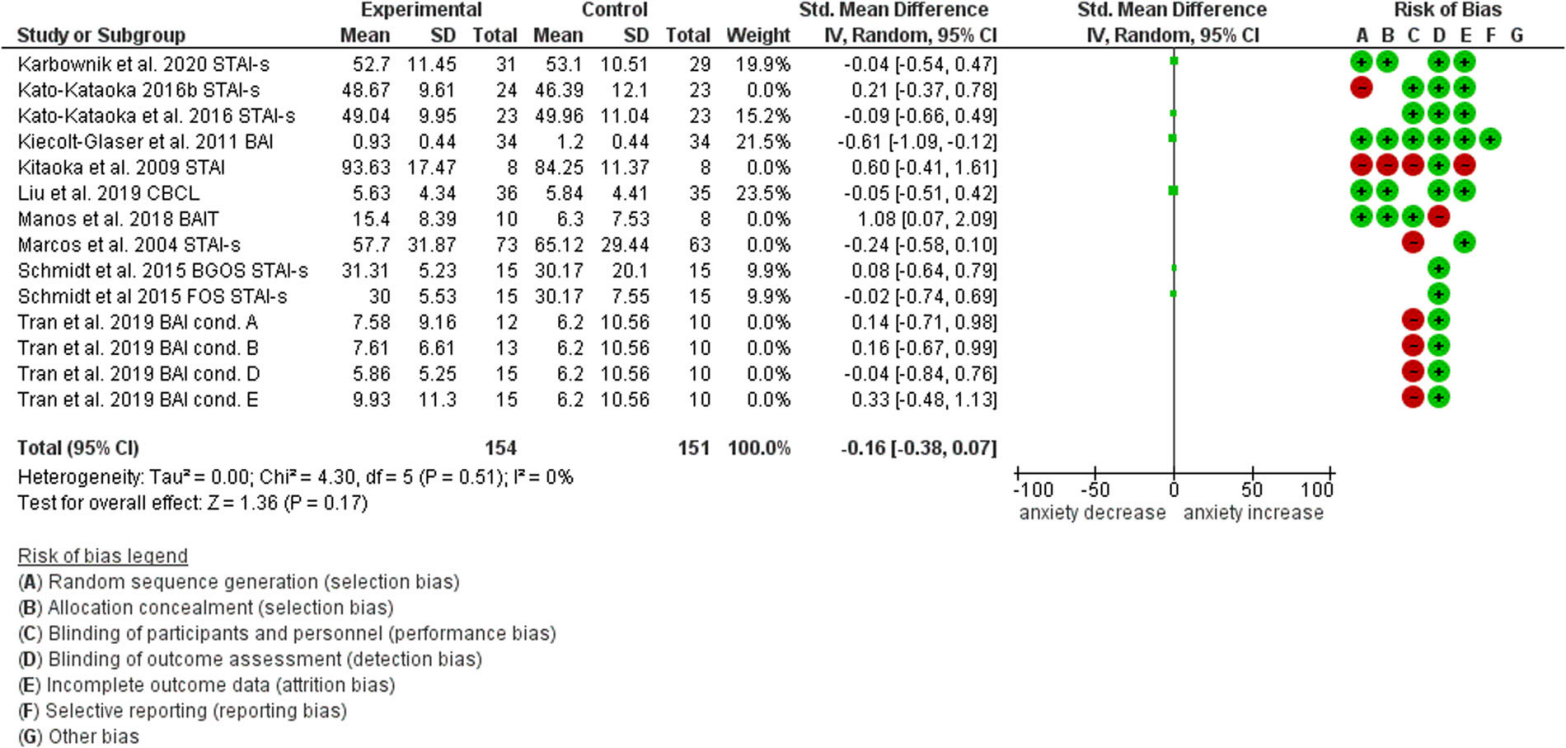
Forest plot excluding the studies at high risk of bias. Reasons for high risk are as follows: **A** Marcos et al. ^33^: concerns in regards to the randomization and allocation sequence and non-blinded design; **B** Kitaoka et al. ^39^: unclear anxiety score differences at baseline between the active and control group, absence of a participants flow diagram and of any relevant information about intervention adherence and missing data; **C** Manos et al. ^36^: unclear anxiety score differences at baseline between active (severe anxiety levels) and control (moderate anxiety levels) groups, not specified reasons for no intervention adherence and missing data, no measurement of state anxiety; **D** Kato-Kataoka et al. ^29^: no randomized allocation, significantly different anxiety scores at baseline (*p* < 0.05) between the active and control group; **E** Tran et al. ^30^: concerns in regard with randomization and allocation process, not enough information about adherence to the intervention and missing data, concerns about the performed statistical analyses.

Of the 14 studies used for the systematic review, 10 were included in the statistical summary (the remaining studies did not assess anxiety but stress only) which was performed with RevMan5. For each study, the standard mean difference between the active and control groups was calculated for continuous anxiety outcomes and a Forest plot created (Fig. 3). Combination of the SMDs revealed a pooled effect size of –0.03 (95% CI: –0.21, 0.14), pointing towards absence of any intervention effect. However, given that the singular SMDs differed substantially amongst each other, ranging from outcomes showing a consistent between-groups anxiety increase^36,39^, as well as a significant decrease^38^, we also performed a sensitivity analysis based on study quality (see Appendices 2, 3, and 4 for the complete risk of bias assessment tables and funnel plot) in order to assess whether any other additional variables other than the intervention might have biased study results. We found that when at high risk of bias studies were excluded, the effect size increased, reaching a value of –0.16 (95% CI: –0.38, 0.07) (Fig. 4). It is worth noting that the only study at low risk of bias was that reporting the highest effect size: –0.61 (95% CI: –1.09, –0.12)^38^, whereas the two studies reporting an increase in anxiety^36,39^ were amongst those at highest risk.

## Limitations

Although informative, the findings of the current systematic review are limited by a couple of factors. First, the small amount of controlled trials done so far within the specific age-range of adolescence and selectively considering clinically (sub)anxious samples. Among them, the presence of several studies at high risk of bias, as outlined in Fig. 3, challenges the reliability of the individual outcomes. Noticeably, most studies lack a pre-specified protocol, a fact that raises some concerns in terms of selective reporting within studies. This, together with our choice to exclude unpublished research, warrants caution in the interpretation of the findings of this systematic review and prevents the drawing of any solid conclusion about the effect of psychobiotics in anxiety during adolescence. However, increasing awareness of these weaknesses will be of help in guiding future research and suggests that more controlled trials are needed—possibly controlling for age range and gender—to allow future systematic reviews and meta-analyses to have sufficient power to detect patterns of responses to psychobiotc interventions.

## Discussion

Based on our systematic review, we conclude that there is currently limited evidence for use of psychobiotics to treat stress and anxiety in youth. As mentioned above, strongest effects may be found in persons with high anxious traits^27^ and therefore, whereas the vast majority of studies in this review used unselected samples, future studies should involve clinical and borderline clinically anxious populations. There is also a need for more high-quality studies which use validated anxiety instruments and behavioural tasks. In particular, it would be important to differentiate whether the intervention aims to improve state and/or trait anxiety, and whether different intervention schedules are required for changing state anxiety as opposed to trait anxiety. Critically, all future studies should include stool sample collections for gut microbial sequencing to assess direct impacts of intervention on the gut microbiome.

The use of psychobiotics to treat anxiety is a research field still very much in its infancy and given the animal literature and some encouraging preliminary findings in humans (albeit not specifically within the age range chosen for this report), combined with the importance of vulnerability to stress in the adolescent developmental window, we suggest that further research could yield inexpensive, safe and effective means to better manage anxiety. To evaluate this perspective, a consultation was carried out with youth with experience of mental health problems, in collaboration with the McPin foundation. This consultation consisted of an online questionnaire of open-ended questions which was completed by 46 participants (mean age = 18.3 years, SD = 2.55 years, 4 males), and a discussion group with 5 young people (aged 14–17 years, 1 male) with lived experience of anxiety and two members of the McPin Foundation. It was found that our view is echoed by young people with lived experience, who not only reported anecdotal evidence of successful psychobiotic interventions, but who also expressed strong interest in contributing to this research drive. Just over two thirds of young people with lived experience of clinical levels of anxiety declared that they had previously attempted to influence mental health and well-being with dietary interventions, such as reducing sugar intake or increasing the amount of daily fruit and vegetables eaten, or introducing supplements, such as probiotics and prebiotics, often following the suggestions of family, friends or a GP. All names have been changed. For example, Jane said:

*“I have anxiety and have been taking probiotics since January for a combo of reasons (gut health, acne, mental health). 100% have noticed a difference and agree that more research in this area is needed”*.

*Mehta added: “I’m really interested in how gut health affects us and I notice it’s positive effects personally too, particularly when switching between eating meat and not eating meat and consumption of sugar”.*

### Young people with lived experience request clearer instructions

Based on the literature, it would appear that specifically, consumption of *L. casei Shirtoa, L. rhamnosus, L. helveticus*, and *Bifidobacterium* (e.g. *B. infantis*) may provide some protection from the anxiogenic effects of environmental stress, and further long-term studies examining whether the development of anxious traits could be partially ameliorated by these probiotics. The consultation with young people noted that such information and guidance on specific bacterial strains would be welcome as the vast number of commercially available probiotic combinations are very confusing. As Becky put it:

*“When I go into* chemist *to buy probiotics, there are millions of products available and the prices vary hugely, so I simply don’t know what to choose and I end up getting nothing. It would really help to receive some clarifications on what actually works”*.

As evident from the review above and the meta-analysis, prebiotics hold particular promise as they are non-digestible (fibre) and thus, unlike some probiotics, reliably arrive fully in the gut. Best bets for further study are GOS and fructooligosaccharides which stimulate growth of beneficial *Lactobacillus* and *Bifidobacterium* species. These compounds are also more stable and not subject to the same degradation in potency over time that can be problematic in probiotics. Prebiotics such as FOS make up part of the dietary fibre in naturally occurring foods such as dairy, or are now widely available in concentrated form as food additives or supplements^40^, which start from around £10 for a month’s supply.

### Psychobiotic interventions could benefit from existing cultural practises

One advantage of psychobiotics in the treatment and prevention of youth anxiety that may have been as yet overlooked is the existing cross-cultural prevalence of fermented food sources containing psychobiotics. For instance, anxiolytic strains of *lactobacilli* have been found in traditional fermented doughs from the Congo and Burkina Faso^41^, Japanese fermented fish^42^, dairy and pickles from China and Mongolia^43^, and eastern European *kefir* fermented milk^44^. This means that the general public might be responsive to a behavioural ‘nudge’, as part of a public health campaign for increasing the anxiolytic psychobiotics consumption for example, given that these products exist widely in many cultures and therefore potentially present fewer cultural barriers to use.

### Young people would like to understand ‘the bigger picture’

The young people in our discussion group showed much enthusiasm and support for more research in the area of dietary interventions, especially in light of the current gap for the age range of 10–24 years. However, they also asked for research approaches that would look at the bigger picture, which could include information on an individual’s personal circumstances and living situation. What was specifically discouraged was yet another prescriptive approach that would require a change in diet, irrespective of other factors such as educational pressure, work environment aspects such as shift work, sleeping patterns, exercise or a number of other, common comorbidities such as depression. Based on our systematic review, we very much agree with the young people and would like to suggest that one possible explanation for the current success of psychobiotic interventions in animal research is that these studies have maximal control over all these different factors through the use of large, longitudinal cohorts and optimal control of environmental factors, whereas human studies either have chosen not to address these potentially confounding variables or been unable to do so.

### Sample-specific considerations for future research

The young people provided a number of important suggestions with regards to new research going forward. For example, when discussing different confounding factors, they stressed repeatedly that any data collection on nutritional intake would need to proceed with caution. Specifically, it was warned that providing participants with numerical feedback, such as total calories consumed per day, would easily trigger attempts to control these numbers, as any potential mechanism of control would be latched upon, with potentially far reaching consequences, such as the risk of developing an eating disorder. The young people conceded that while it would be important to obtain comprehensive data on nutritional intake and dietary habits, it would be more helpful to withhold specific detailed feedback and only provide general pointers about nutritional health for example. Last, the young people stated that providing faeces for microbiome sequencing could be a real obstacle for this kind of research, but that they would consider participating if there were a number of options available for stool sampling (i.e. at home or at a testing centre), and that discretion (e.g. packaging for the stool sample that would be both leak and smell-proof, as well as neutrally designed) was paramount. These are all very valuable points as both nutritional analyses, as well as microbiome sequencing are both cornerstones of dietary intervention research into the microbiome gut–brain axis.

### Towards a new, multidisciplinary research approach

Going forward, it is our view that in order to future-proof this new research area and to allow for sustained scientific progress and breakthrough, what is now needed are systematic, multidisciplinary approaches that consider not only the effect of the dietary intervention on composition of the microbiome, but also on how interventions interact with ongoing brain maturation and functional responsiveness^45,46^ (see also ref. ^46^ for an extended discussion). Similarly, and in line with the important points highlighted by the young people in our consultation, other factors such as hormonal changes due to the specific puberty stage or menstrual cycle, sleep hygiene and patterns, as well as life-style factors such as nutrition and exercise will need to be included to obtain a comprehensive, ‘bigger picture’. Such an approach would also increase uptake and compliance with any future interventions.

Future studies could adopt a research approach that is already practised in the field of developmental cognitive neuroscience (DCN)^45^, which focuses on investigating how the complex interplay of genetic, environmental and brain maturational factors shape psychological functioning in development to improve outcomes for the individual^47–49^. Moreover, placed at the intersection of nature versus nurture, the DCN research approach always assumes a multi-level and multi-factor approach to understanding change which, by definition is multidisciplinary. Given that the field of microbiome and gut–brain axis research is still emerging and finding its shape, we would like to stress that any real progress will depend on the adoption of a similarly comprehensive multifactorial and multidisciplinary research approach for pinpointing mechanisms and translation in both animal and human models. Therefore, priority for funding should be given to projects that bring together the expertise and the collaboration of scientists from a range of fields (education, psychology, microbiology, neuroscience and nutrition) and, importantly, guidance and advice from young people with lived experience to ensure that all research continues to address the questions that are relevant to the lives of young people with anxiety.

## Conclusion

To conclude, the gut microbiome, and its effect on behaviour and mental health has captured the interest and imagination of scientists and the wider public alike. However, as a still relatively unexplored area, any real progress will require a systematic multidisciplinary research approach, which gives priority to specifying mechanisms in the human and animal models, providing causal understanding and addressing realistic outcomes. This is particularly critical in light of strong public and commercial interests that are presently outpacing research efforts. Encouragingly, this approach has also been met with much enthusiasm from young people with lived experience of mental health problems, which suggests that these research approach and interventions may be ripe for a behavioural ‘nudge’ and potentially present few cultural barriers to use.

## Supplementary information

SM Psychobiotic interventions for anxiety in young people

## Data Availability

Data are available from the corresponding author on reasonable request.
