# Supplementary material for: Psychobiotic interventions for anxiety in young people: a systematic review and meta-analysis, with youth consultation
Source: Transl Psychiatry. 2021 Jun 16;11:352. doi: 10.1038/s41398-021-01422-7 (PMC8206413; doi:10.1038/s41398-021-01422-7)
Supplement: Supplementary file 1 — SM Psychobiotic interventions for anxiety in young people [file 41398_2021_1422_MOESM1_ESM.docx]

**Appendix 1:** search terms

**PUBMED**: 449 results

#1 humans[MeSH Terms] OR patients[MeSH Terms] OR research subjects[MeSH Terms] OR human experimentation[MeSH Terms] OR human*[title/abstract] OR client*[title/abstract] OR individual*[title/abstract] OR subject*[title/abstract] OR participant* [title/abstract

#2 minors[MeSH Terms] OR adolescent[MeSH Terms] OR child[MeSH Terms] OR adolescence[MeSH Terms] OR adolescent, development[MeSH Terms] OR child, development[MeSH Terms] OR paediatric[MeSH Terms] OR puberty[Mesh Terms] OR young adult[sMeSH Terms] OR teen*[title/abstract] OR young men[title/abstract] OR young women[title/abstract] OR adolescen*[title/abstract] OR youth [title/abstract] OR undergraduate student* [title/abstract] OR college student* [title/abstract]

#3 prebiotics[MeSH Terms] OR probiotics[MeSH Terms] OR dietary carbohydrates[Mesh Terms OR dietary fiber[MeSH Terms] OR psychobiotic*[title/abstract] OR probio*[title/abstract] OR prebio*[title/abstract] OR pro-bio*[title/abstract] OR pre-bio*[title/abstract] OR lactobacill* [title/abstract] OR bifidobacteri*[title/abstract]

#4 anxiety[MeSH Terms] OR anxiety disorders[MeSH Terms] OR test anxiety scale[MeSH Terms] OR manifest anxiety scale[MeSH Terms] OR patient health questionnaire[MeSH Terms] OR survey and questionnaire/psychology[MeSH Terms] OR psychology, child[MeSH Terms] OR psychology, adolescent[MeSH Terms] OR child behavior disorders[MeSH Terms] OR panic[MeSH Terms] OR affect[Mesh Terms] OR affective symptoms[MeSH Terms] OR performance anxiety[MeSH Terms] OR stress, psychological[MeSH Terms] OR anxi*[title/abstract] OR stress[Text Word] OR PSS[Text Word] OR SAS[Text Word] OR DASS[Text Word] OR HADS[Text Word] OR HAD-S[Text Word] OR BAI[Text Word] OR STAI[Text Word] OR PHQ[Text Word] OR Emotional decision*[text word] OR psychological distress [mesh terms] OR emotions[mesh Terms] OR emotion regulation [mesh terms] OR "emotional stroop" [text word] OR "dot probe[text word]

**COCHRANE**: 372 results

#1 [mh humans] OR [mh patients] or [mh "research subjects"] OR [mh "human experimentation"] OR participant OR client OR individual OR subject

#2 [mh minors] OR [mh adolescent] OR [mh adolescence] OR [mh child] or [mh puberty] or [mh "adolescent, development"] or [mh "child, development"] OR [mh paediatric] or "young men" or "young women" or teen* or adolescen* or “undergraduate student” or “college student”

#3 [mh probiotics] or [mh prebiotics] or psychobiotic* or probio* or prebio* or [mh "dietary carbohydrates"] or [mh "dietary fiber"] or bifidobacteri* or lactobacill*

#4 [mh anxiety] or [mh "anxiety disorders"] or [mh affect] or [mh panic] or [mh "affective symptoms"] or [mh "patient health questionnaire"] or [mh "survey and questionnaires"/PX] or [mh "psychology, child"] or [mh "psychology, adolescent"] or [mh "test anxiety scale"] or [mh "manifest anxiety scale"] or [mh "child behavior disorders"] or [mh "performance anxiety"] or [mh "stress, psychological"] or [mh "emotion regulation"] or [mh emotions] or [mh "psychological distress"] or anxi* or sas or dass or hads or had-s or stai or bai or phq or pss or "emotional stroop" or "dot-probe"

**MEDLINE**: 105 results

#1 (MM "Humans") OR (MH "Patients+") OR (MH "Research Subjects+") OR (MH "Human Experimentation+") OR "human*" OR "subject*" OR "individual* OR "client*" OR participant*

#2 (MM "Minors") OR (MM "Adolescent") OR (MM "Adolescent Development") OR (MH "Child+") OR (MH "Child Development+") OR "adolescen*" OR (MH "Pediatrics") OR (MM "Puberty") OR (MM "Young Adult") OR "teen*" OR "youth" OR “young men” OR “young women” or “undergraduate student” or “college student”

#3 (MM "Probiotics") OR (MM "Prebiotics") OR (MM "Dietary Carbohydrates") OR (MM "Dietary Fiber") OR "psychobiotic*" OR "probio* OR "prebio*" OR "pro-bio*" OR "pre-bio*" OR “lactobacill*” OR “bifidobacteri*”

#4 (MH "Anxiety+") OR (MH "Anxiety Disorders+") OR (MM "Test Anxiety Scale") OR (MM "Manifest Anxiety Scale") OR (MM "Patient Health Questionnaire") OR (MM "Surveys and Questionnaires”/PX) OR (MM "Psychology, Child") OR (MM "Psychology, Adolescent") OR (MM "Child Behavior Disorders") OR (MM "Panic") OR (MH "Affect+") OR (MH "Affective Symptoms+") OR (MM "Performance Anxiety") OR (MH "Stress, Psychological+") OR (MH "Emotions+") OR (MH "Psychological Distress") OR (MM "Emotional Regulation") OR "SAS" OR "DASS" OR "HADS" OR "HAD-S" OR "BAI" OR "STAI" OR "PHQ" OR "PSS" OR "anxi*" OR “emotional stroop” OR "Emotional decision*” OR “dot probe" OR "stress"

**SCOPUS**:2071 results

((TITLE-ABS-KEY(human) OR TITLE-ABS-KEY(patient) OR TITLE-ABS-KEY("research subject") OR TITLE-ABS-KEY("human trial") OR TITLE-ABS-KEY(subject) OR TITLE-ABS-KEY(client) OR TITLE-ABS-KEY(individual))) and ((TITLE-ABS-KEY(minor) OR TITLE-ABS-KEY(adolescen*) OR TITLE-ABS-KEY(child) OR TITLE-ABS-KEY("young adult") OR TITLE-ABS-KEY(teen*) OR TITLE-ABS-KEY("young m?n") OR TITLE-ABS-KEY("young wom?n") OR TITLE-ABS-KEY(youth) OR TITLE-ABS-KEY("undergraduate student*") OR TITLE-ABS-KEY("college student*"))) and ((TITLE-ABS-KEY(prebiotic) OR TITLE-ABS-KEY(probiotic) OR TITLE-ABS-KEY(psychobiotic) OR TITLE-ABS-KEY(lactobacill*) OR TITLE-ABS-KEY(bifidobacter*) OR TITLE-ABS-KEY("dietary fiber"))) and ((TITLE-ABS-KEY(anxi*) OR TITLE-ABS-KEY(affect*) OR TITLE-ABS-KEY("emotion regulation") OR TITLE-ABS-KEY("emotional disorder") OR TITLE-ABS-KEY("patient health questionnaire") OR TITLE-ABS-KEY(panic) OR TITLE-ABS-KEY(psychologic*) OR TITLE-ABS-KEY(sas) OR TITLE-ABS-KEY(dass) OR TITLE-ABS-KEY(had?s) OR TITLE-ABS-KEY(bai) OR TITLE-ABS-KEY(stai) OR TITLE-ABS-KEY(pss) OR TITLE-ABS-KEY(phq) OR TITLE-ABS-KEY("emotional stroop") OR TITLE-ABS-KEY("dot?probe") OR TITLE-ABS-KEY(stress) OR TITLE-ABS-KEY(distress)))

**EMBASE**: 347 results

#1 'human'/exp OR 'patient'/exp OR 'research subject'/exp OR 'human experiment'/exp OR human*:ti,ab OR client*:ti,ab OR individual*:ti,ab OR subject*:ti,ab OR participant*:ti,ab

#2 'minor'/exp OR 'adolescent'/exp OR 'child'/exp OR 'adolescence'/exp OR 'young adult'/exp OR 'young m#n':ti,ab OR 'young wom#n':ti,ab OR teen*:ti,ab OR adolescen*:ti,ab OR youth:ti,ab or ‘undergraduate student*’:ti,ab or ‘college student*’:ti,ab

#3 'probiotic agent'/exp OR 'prebiotic agent'/exp OR 'dietary fiber'/exp OR 'psychobiotic agent'/exp OR 'lactobacillus'/exp OR 'bifidobacterium'/exp OR psychobiotic*:ti,ab OR pro?bio*:ti,ab OR pre?bio*:ti,ab OR lactobacill*:ti,ab OR bifidofacteri*:ti,ab

#4 'anxiety'/exp OR 'anxiety disorder'/exp OR 'anxiety psychology'/exp OR 'anxiety assessment'/exp OR 'child psychology'/exp OR 'affect'/exp OR 'emotion regulation'/exp OR 'emotional disorder'/exp OR 'emotional stress'/exp OR 'patient health questionnaire'/exp OR 'behavior/psychological aspect' OR sas:ti,ab OR dass:ti,ab OR had?s:ti,ab OR bai:ti,ab OR stai:ti,ab OR phq:ti,ab OR pss:ti,ab OR anxi*:ti,ab OR 'emotional stroop':ti,ab OR 'dot?probe':ti,ab OR distress:ti,ab OR stress:ti,ab OR 'emotional decision*':ti,ab

**PSYCHINFO**: 33 results

#1 DE "Experimental Subjects" OR AB participant* OR TABsubject* OR AB individual* OR AB human*

#2 DE "Adolescent Psychopathology" OR DE "Adolescent Psychology" OR DE "Adolescent Psychiatry" OR DE "Adolescent Development" OR DE "Adolescent Behavior" OR DE "Adolescent Characteristics" OR DE "Adolescent Health" OR DE "Early Adolescence" OR AB child* OR AB adolescen* OR AB teen* OR AB youth OR AB "young adult*" OR AB "young women" OR AB "young men" or AB“undergraduate student*” or AB“college student*”

#3 DE "Dietary Supplements" OR AB probio* OR AB prebio* OR AB lactobacill* OR AB bifidobacteri* OR AB psychobiotic* OR AB “dietary fiber*”

#4 ((((((((((DE "Anxiety" OR DE "Anxiety Sensitivity" OR DE "Computer Anxiety" OR DE "Health Anxiety" OR DE "Mathematics Anxiety" OR DE "Performance Anxiety" OR DE "Social Anxiety" OR DE "Speech Anxiety" OR DE "Test Anxiety" OR DE "Anxiety Disorders" OR DE "Castration Anxiety" OR DE "Death Anxiety" OR DE "Generalized Anxiety Disorder" OR DE "Obsessive Compulsive Disorder" OR DE "Panic Attack" OR DE "Panic Disorder" OR DE "Phobias" OR DE "Separation Anxiety Disorder" OR DE "Trichotillomania" OR DE "Taylor Manifest Anxiety Scale" OR DE "State Trait Anxiety Inventory" OR DE "Childrens Manifest Anxiety Scale") OR (DE "Child Behavior Checklist")) OR (DE "Mental Health and Illness Assessment")) OR (DE "Health Psychology Assessment" OR DE "Health Attitude Measures" OR DE "Health Behavior Measures"))) OR (DE "Psychodiagnostic Measures")) OR (DE "Psychodiagnostic Interview" OR DE "Diagnostic Interview Schedule" OR DE "Structured Clinical Interview")) OR (DE "Attentional Bias")) OR (DE "Behavioral Assessment")) OR (DE "Distress") OR AB "emotion regulation" OR TX "emotional stroop" OR TX "dot probe" OR TX SAS OR TX DASS OR TX DA?S OR TX BAI OR TX STAI OR TX PHQ OR TX PSS

**WEB OF SCIENCE**: 2029 results

#1 TOPIC (human* OR patient* OR subject* OR client* OR individual*)

#2 TOPIC (adolescen* OR child* OR "young adult*"OR teen* OR "young m?n" OR "young wom?n" OR youth or “undergraduate student*” or “college student*”)

#3 TOPIC (prebiotic* OR probiotic* OR psychobiotic*OR lactobacill* OR bifidobacter*OR "dietary fiber*")

#4 TOPIC (anxi* OR affect*OR "emotion* regulation" OR "emotional disorder* OR "patient health questionnaire" OR sas OR dass OR had?s OR stai OR bai OR phq OR pss OR panicOR "emotional stroop"OR " dot?probe" OR "psychological distress" OR stress)

**Appendix 2: Risk of Bias Assessment of anxiety studies**

| **Unique ID** | K20STAIS | **Study ID** | Kar20 | **Assessor** |  |
| --- | --- | --- | --- | --- | --- |
| **Ref or Label** | Karbownik 2020 | **Aim** | assignment to intervention (the 'intention-to-treat' effect) |  |  |
| **Experimental** | Probiotic | **Comparator** | placebo | **Source** |  |
| **Outcome** | STAI state | **Results** | -0,04 | **Weight** | 19.9 |
| **Domain** | **Signalling question** | | | **Response** | **Comments** |
| **Bias arising from the randomization process** | 1.1 Was the allocation sequence random? | | | Y |  |
|  | 1.2 Was the allocation sequence concealed until participants were enrolled and assigned to interventions? | | | Y |  |
|  | 1.3 Did baseline differences between intervention groups suggest a problem with the randomization process? | | | N |  |
|  | **Risk of bias judgement** | | | **Low** |  |
| **Bias due to deviations from intended interventions** | 2.1.Were participants aware of their assigned intervention during the trial? | | | N |  |
|  | 2.2.Were carers and people delivering the interventions aware of participants' assigned intervention during the trial? | | | N |  |
|  | 2.3. If Y/PY/NI to 2.1 or 2.2: Were there deviations from the intended intervention that arose because of the experimental context? | | | PN |  |
|  | 2.4 If Y/PY to 2.3: Were these deviations likely to have affected the outcome? | | | NA |  |
|  | 2.5. If Y/PY/NI to 2.4: Were these deviations from intended intervention balanced between groups? | | | NA |  |
|  | 2.6 Was an appropriate analysis used to estimate the effect of assignment to intervention? | | | PY |  |
|  | 2.7 If N/PN/NI to 2.6: Was there potential for a substantial impact (on the result) of the failure to analyse participants in the group to which they were randomized? | | | PN |  |
|  | **Risk of bias judgement** | | | **Some concerns** |  |
| **Bias due to missing outcome data** | 3.1 Were data for this outcome available for all, or nearly all, participants randomized? | | | N |  |
|  | 3.2 If N/PN/NI to 3.1: Is there evidence that result was not biased by missing outcome data? | | | PY | ITT analysis did not show different statistical results in comparison to PP analysis |
|  | 3.3 If N/PN to 3.2: Could missingness in the outcome depend on its true value? | | | NA |  |
|  | 3.4 If Y/PY/NI to 3.3: Is it likely that missingness in the outcome depended on its true value? | | | NA |  |
|  | **Risk of bias judgement** | | | **Low** |  |
| **Bias in measurement of the outcome** | 4.1 Was the method of measuring the outcome inappropriate? | | | N |  |
|  | 4.2 Could measurement or ascertainment of the outcome have differed between intervention groups? | | | N |  |
|  | 4.3 Were outcome assessors aware of the intervention received by study participants? | | | N |  |
|  | 4.4 If Y/PY/NI to 4.3: Could assessment of the outcome have been influenced by knowledge of intervention received? | | | NA |  |
|  | 4.5 If Y/PY/NI to 4.4: Is it likely that assessment of the outcome was influenced by knowledge of intervention received? | | | NA |  |
|  | **Risk of bias judgement** | | | **Low** |  |
| **Bias in selection of the reported result** | 5.1 Were the data that produced this result analysed in accordance with a pre-specified analysis plan that was finalized before unblinded outcome data were available for analysis? | | | NI |  |
|  | 5.2 ... multiple eligible outcome measurements (e.g. scales, definitions, time points) within the outcome domain? | | | NI |  |
|  | 5.3 ... multiple eligible analyses of the data? | | | NI |  |
|  | **Risk of bias judgement** | | | **Some concerns** |  |
| **Overall bias** | **Risk of bias judgement** | | | **Some concerns** |  |
|  |  |  |  |  |  |
|  |  |  |  |  |  |
| **Unique ID** | K09STAITOT | **Study ID** | Kit09 | **Assessor** |  |
| **Ref or Label** | Kitaoka 2009 | **Aim** | assignment to intervention (the 'intention-to-treat' effect) |  |  |
| **Experimental** | prebiotic | **Comparator** | placebo | **Source** |  |
| **Outcome** | STAI-total | **Results** | 0,6 | **Weight** | 2.8 |
| **Domain** | **Signalling question** | | | **Response** | **Comments** |
| **Bias arising from the randomization process** | 1.1 Was the allocation sequence random? | | | NI |  |
|  | 1.2 Was the allocation sequence concealed until participants were enrolled and assigned to interventions? | | | NI |  |
|  | 1.3 Did baseline differences between intervention groups suggest a problem with the randomization process? | | | PY | prebiotic group: 100.5 at baseline down to 93.63 at t2 (with p<0.05)  placebo group: 87.73 at baseline down to 84.25 |
|  | **Risk of bias judgement** | | | **High** |  |
| **Bias due to deviations from intended interventions** | 2.1.Were participants aware of their assigned intervention during the trial? | | | N |  |
|  | 2.2.Were carers and people delivering the interventions aware of participants' assigned intervention during the trial? | | | N |  |
|  | 2.3. If Y/PY/NI to 2.1 or 2.2: Were there deviations from the intended intervention that arose because of the experimental context? | | | NI |  |
|  | 2.4 If Y/PY to 2.3: Were these deviations likely to have affected the outcome? | | | NA |  |
|  | 2.5. If Y/PY/NI to 2.4: Were these deviations from intended intervention balanced between groups? | | | NA |  |
|  | 2.6 Was an appropriate analysis used to estimate the effect of assignment to intervention? | | | NI |  |
|  | 2.7 If N/PN/NI to 2.6: Was there potential for a substantial impact (on the result) of the failure to analyse participants in the group to which they were randomized? | | | NI |  |
|  | **Risk of bias judgement** | | | **High** | The authors did not include a participant flow diagram or any relevant information about adherence to intervention or protocol deviations |
| **Bias due to missing outcome data** | 3.1 Were data for this outcome available for all, or nearly all, participants randomized? | | | NI |  |
|  | 3.2 If N/PN/NI to 3.1: Is there evidence that result was not biased by missing outcome data? | | | PN |  |
|  | 3.3 If N/PN to 3.2: Could missingness in the outcome depend on its true value? | | | NI |  |
|  | 3.4 If Y/PY/NI to 3.3: Is it likely that missingness in the outcome depended on its true value? | | | NI |  |
|  | **Risk of bias judgement** | | | **High** | Again, authors did not provide a flow diagram, or give detailed information about participants who entered the studies, lost to follow-up, withdrawn, etc. |
| **Bias in measurement of the outcome** | 4.1 Was the method of measuring the outcome inappropriate? | | | N |  |
|  | 4.2 Could measurement or ascertainment of the outcome have differed between intervention groups? | | | N |  |
|  | 4.3 Were outcome assessors aware of the intervention received by study participants? | | | N |  |
|  | 4.4 If Y/PY/NI to 4.3: Could assessment of the outcome have been influenced by knowledge of intervention received? | | | NA |  |
|  | 4.5 If Y/PY/NI to 4.4: Is it likely that assessment of the outcome was influenced by knowledge of intervention received? | | | NA |  |
|  | **Risk of bias judgement** | | | **Low** |  |
| **Bias in selection of the reported result** | 5.1 Were the data that produced this result analysed in accordance with a pre-specified analysis plan that was finalized before unblinded outcome data were available for analysis? | | | NI |  |
|  | 5.2 ... multiple eligible outcome measurements (e.g. scales, definitions, time points) within the outcome domain? | | | NI |  |
|  | 5.3 ... multiple eligible analyses of the data? | | | NI |  |
|  | **Risk of bias judgement** | | | **Some concerns** |  |
| **Overall bias** | **Risk of bias judgement** | | | **High** | The authors did not give enough information about the randomization and allocation process. Anxiety scores of the two groups differed at baseline, possibly biasing the comparison between group at t2 as no baseline correction has been carried out. Indeed, significant decrease in anxiety scores was found in the prebiotic group (<0,05) only, but the comparison between placebo and experimental group means at t2 does not reflect such effect (d=0,6). Finally, the authors did not provide a participant flow diagram or information about adherence and missing data. |
|  |  |  |  |  |  |
|  |  |  |  |  |  |
| **Unique ID** | K11BAI | **Study ID** | Kie11 | **Assessor** |  |
| **Ref or Label** | Kiecolt-Glaser 2011 | **Aim** | assignment to intervention (the 'intention-to-treat' effect) |  |  |
| **Experimental** | prebiotic | **Comparator** | placebo | **Source** | Non-commercial trial registry record (e.g. ClinicalTrials.gov record) |
| **Outcome** | BAI | **Results** | -0,61 | **Weight** | 10.6 |
| **Domain** | **Signalling question** | | | **Response** | **Comments** |
| **Bias arising from the randomization process** | 1.1 Was the allocation sequence random? | | | Y |  |
|  | 1.2 Was the allocation sequence concealed until participants were enrolled and assigned to interventions? | | | Y |  |
|  | 1.3 Did baseline differences between intervention groups suggest a problem with the randomization process? | | | PN |  |
|  | **Risk of bias judgement** | | | **Low** |  |
| **Bias due to deviations from intended interventions** | 2.1.Were participants aware of their assigned intervention during the trial? | | | N |  |
|  | 2.2.Were carers and people delivering the interventions aware of participants' assigned intervention during the trial? | | | N |  |
|  | 2.3. If Y/PY/NI to 2.1 or 2.2: Were there deviations from the intended intervention that arose because of the experimental context? | | | NA |  |
|  | 2.4 If Y/PY to 2.3: Were these deviations likely to have affected the outcome? | | | NA |  |
|  | 2.5. If Y/PY/NI to 2.4: Were these deviations from intended intervention balanced between groups? | | | NA |  |
|  | 2.6 Was an appropriate analysis used to estimate the effect of assignment to intervention? | | | Y |  |
|  | 2.7 If N/PN/NI to 2.6: Was there potential for a substantial impact (on the result) of the failure to analyse participants in the group to which they were randomized? | | | NA |  |
|  | **Risk of bias judgement** | | | **Low** |  |
| **Bias due to missing outcome data** | 3.1 Were data for this outcome available for all, or nearly all, participants randomized? | | | Y |  |
|  | 3.2 If N/PN/NI to 3.1: Is there evidence that result was not biased by missing outcome data? | | | NA |  |
|  | 3.3 If N/PN to 3.2: Could missingness in the outcome depend on its true value? | | | NA |  |
|  | 3.4 If Y/PY/NI to 3.3: Is it likely that missingness in the outcome depended on its true value? | | | NA |  |
|  | **Risk of bias judgement** | | | **Low** |  |
| **Bias in measurement of the outcome** | 4.1 Was the method of measuring the outcome inappropriate? | | | N |  |
|  | 4.2 Could measurement or ascertainment of the outcome have differed between intervention groups? | | | N |  |
|  | 4.3 Were outcome assessors aware of the intervention received by study participants? | | | N |  |
|  | 4.4 If Y/PY/NI to 4.3: Could assessment of the outcome have been influenced by knowledge of intervention received? | | | NA |  |
|  | 4.5 If Y/PY/NI to 4.4: Is it likely that assessment of the outcome was influenced by knowledge of intervention received? | | | NA |  |
|  | **Risk of bias judgement** | | | **Low** |  |
| **Bias in selection of the reported result** | 5.1 Were the data that produced this result analysed in accordance with a pre-specified analysis plan that was finalized before unblinded outcome data were available for analysis? | | | NI | The trial protocol pre-specifies how the outcomes will be treated but not how statistically analysed |
|  | 5.2 ... multiple eligible outcome measurements (e.g. scales, definitions, time points) within the outcome domain? | | | N |  |
|  | 5.3 ... multiple eligible analyses of the data? | | | N |  |
|  | **Risk of bias judgement** | | | **Low** | In the paper authors mentioned pre-planned analysis to be in line with the performed analysis. |
| **Overall bias** | **Risk of bias judgement** | | | **Low** |  |
|  |  |  |  |  |  |
|  |  |  |  |  |  |
| **Unique ID** | L188CBCL | **Study ID** | Liu19 | **Assessor** |  |
| **Ref or Label** | Liu 2019 | **Aim** | assignment to intervention (the 'intention-to-treat' effect) |  |  |
| **Experimental** | probiotic | **Comparator** | placebo | **Source** | Non-commercial trial registry record (e.g. ClinicalTrials.gov record) |
| **Outcome** | CBCL | **Results** | -0,05 | **Weight** | 11.4 |
| **Domain** | **Signalling question** | | | **Response** | **Comments** |
| **Bias arising from the randomization process** | 1.1 Was the allocation sequence random? | | | Y |  |
|  | 1.2 Was the allocation sequence concealed until participants were enrolled and assigned to interventions? | | | Y |  |
|  | 1.3 Did baseline differences between intervention groups suggest a problem with the randomization process? | | | N |  |
|  | **Risk of bias judgement** | | | **Low** |  |
| **Bias due to deviations from intended interventions** | 2.1.Were participants aware of their assigned intervention during the trial? | | | N |  |
|  | 2.2.Were carers and people delivering the interventions aware of participants' assigned intervention during the trial? | | | N |  |
|  | 2.3. If Y/PY/NI to 2.1 or 2.2: Were there deviations from the intended intervention that arose because of the experimental context? | | | N |  |
|  | 2.4 If Y/PY to 2.3: Were these deviations likely to have affected the outcome? | | | NA |  |
|  | 2.5. If Y/PY/NI to 2.4: Were these deviations from intended intervention balanced between groups? | | | NA |  |
|  | 2.6 Was an appropriate analysis used to estimate the effect of assignment to intervention? | | | N | Participants who discontinued the intervention were excluded from the analysis |
|  | 2.7 If N/PN/NI to 2.6: Was there potential for a substantial impact (on the result) of the failure to analyse participants in the group to which they were randomized? | | | PN |  |
|  | **Risk of bias judgement** | | | **Some concerns** |  |
| **Bias due to missing outcome data** | 3.1 Were data for this outcome available for all, or nearly all, participants randomized? | | | N |  |
|  | 3.2 If N/PN/NI to 3.1: Is there evidence that result was not biased by missing outcome data? | | | N |  |
|  | 3.3 If N/PN to 3.2: Could missingness in the outcome depend on its true value? | | | PN |  |
|  | 3.4 If Y/PY/NI to 3.3: Is it likely that missingness in the outcome depended on its true value? | | | NA |  |
|  | **Risk of bias judgement** | | | **Low** |  |
| **Bias in measurement of the outcome** | 4.1 Was the method of measuring the outcome inappropriate? | | | N |  |
|  | 4.2 Could measurement or ascertainment of the outcome have differed between intervention groups? | | | N |  |
|  | 4.3 Were outcome assessors aware of the intervention received by study participants? | | | N |  |
|  | 4.4 If Y/PY/NI to 4.3: Could assessment of the outcome have been influenced by knowledge of intervention received? | | | NA |  |
|  | 4.5 If Y/PY/NI to 4.4: Is it likely that assessment of the outcome was influenced by knowledge of intervention received? | | | NA |  |
|  | **Risk of bias judgement** | | | **Low** |  |
| **Bias in selection of the reported result** | 5.1 Were the data that produced this result analysed in accordance with a pre-specified analysis plan that was finalized before unblinded outcome data were available for analysis? | | | NI | Tn the trial protocol there is no pre-specified analysis information |
|  | 5.2 ... multiple eligible outcome measurements (e.g. scales, definitions, time points) within the outcome domain? | | | N |  |
|  | 5.3 ... multiple eligible analyses of the data? | | | NI |  |
|  | **Risk of bias judgement** | | | **Some concerns** |  |
| **Overall bias** | **Risk of bias judgement** | | | **Some concerns** |  |
|  |  |  |  |  |  |
|  |  |  |  |  |  |
| **Unique ID** | M18BAIT | **Study ID** | Man18 | **Assessor** |  |
| **Ref or Label** | Manos 2018 | **Aim** | assignment to intervention (the 'intention-to-treat' effect) |  |  |
| **Experimental** | prebiotic | **Comparator** | placebo | **Source** |  |
| **Outcome** | BAIT | **Results** | 1,08 | **Weight** | 2.8 |
| **Domain** | **Signalling question** | | | **Response** | **Comments** |
| **Bias arising from the randomization process** | 1.1 Was the allocation sequence random? | | | Y |  |
|  | 1.2 Was the allocation sequence concealed until participants were enrolled and assigned to interventions? | | | Y |  |
|  | 1.3 Did baseline differences between intervention groups suggest a problem with the randomization process? | | | PY | Mean trait anxiety scores are higher in the omega-3 PUFA group (severe anxiety) compared to the placebo group (moderate anxiety) |
|  | **Risk of bias judgement** | | | **Some concerns** |  |
| **Bias due to deviations from intended interventions** | 2.1.Were participants aware of their assigned intervention during the trial? | | | N |  |
|  | 2.2.Were carers and people delivering the interventions aware of participants' assigned intervention during the trial? | | | N |  |
|  | 2.3. If Y/PY/NI to 2.1 or 2.2: Were there deviations from the intended intervention that arose because of the experimental context? | | | NA |  |
|  | 2.4 If Y/PY to 2.3: Were these deviations likely to have affected the outcome? | | | NA |  |
|  | 2.5. If Y/PY/NI to 2.4: Were these deviations from intended intervention balanced between groups? | | | NA |  |
|  | 2.6 Was an appropriate analysis used to estimate the effect of assignment to intervention? | | | Y |  |
|  | 2.7 If N/PN/NI to 2.6: Was there potential for a substantial impact (on the result) of the failure to analyse participants in the group to which they were randomized? | | | NA |  |
|  | **Risk of bias judgement** | | | **Low** |  |
| **Bias due to missing outcome data** | 3.1 Were data for this outcome available for all, or nearly all, participants randomized? | | | N |  |
|  | 3.2 If N/PN/NI to 3.1: Is there evidence that result was not biased by missing outcome data? | | | N |  |
|  | 3.3 If N/PN to 3.2: Could missingness in the outcome depend on its true value? | | | NI |  |
|  | 3.4 If Y/PY/NI to 3.3: Is it likely that missingness in the outcome depended on its true value? | | | PN |  |
|  | **Risk of bias judgement** | | | **Some concerns** |  |
| **Bias in measurement of the outcome** | 4.1 Was the method of measuring the outcome inappropriate? | | | PY | State anxiety should have been measured as well, as more sensible to potential transient reactions related to the intervention |
|  | 4.2 Could measurement or ascertainment of the outcome have differed between intervention groups? | | | N |  |
|  | 4.3 Were outcome assessors aware of the intervention received by study participants? | | | NA |  |
|  | 4.4 If Y/PY/NI to 4.3: Could assessment of the outcome have been influenced by knowledge of intervention received? | | | NA |  |
|  | 4.5 If Y/PY/NI to 4.4: Is it likely that assessment of the outcome was influenced by knowledge of intervention received? | | | NA |  |
|  | **Risk of bias judgement** | | | **High** |  |
| **Bias in selection of the reported result** | 5.1 Were the data that produced this result analysed in accordance with a pre-specified analysis plan that was finalized before unblinded outcome data were available for analysis? | | | NI |  |
|  | 5.2 ... multiple eligible outcome measurements (e.g. scales, definitions, time points) within the outcome domain? | | | NI |  |
|  | 5.3 ... multiple eligible analyses of the data? | | | NI |  |
|  | **Risk of bias judgement** | | | **Some concerns** |  |
| **Overall bias** | **Risk of bias judgement** | | | **High** |  |
|  |  |  |  |  |  |
|  |  |  |  |  |  |
| **Unique ID** | M04STAIS | **Study ID** | Marco04 | **Assessor** |  |
| **Ref or Label** | Marcos 2004 | **Aim** | assignment to intervention (the 'intention-to-treat' effect) |  |  |
| **Experimental** | probiotic | **Comparator** | placebo | **Source** |  |
| **Outcome** | STAI-state | **Results** | -0,24 | **Weight** | 18.6 |
| **Domain** | **Signalling question** | | | **Response** | **Comments** |
| **Bias arising from the randomization process** | 1.1 Was the allocation sequence random? | | | NI |  |
|  | 1.2 Was the allocation sequence concealed until participants were enrolled and assigned to interventions? | | | NI |  |
|  | 1.3 Did baseline differences between intervention groups suggest a problem with the randomization process? | | | N |  |
|  | **Risk of bias judgement** | | | **Some concerns** |  |
| **Bias due to deviations from intended interventions** | 2.1.Were participants aware of their assigned intervention during the trial? | | | PY | The study design is not described as blinded/double blinded. |
|  | 2.2.Were carers and people delivering the interventions aware of participants' assigned intervention during the trial? | | | PY |  |
|  | 2.3. If Y/PY/NI to 2.1 or 2.2: Were there deviations from the intended intervention that arose because of the experimental context? | | | N |  |
|  | 2.4 If Y/PY to 2.3: Were these deviations likely to have affected the outcome? | | | NA |  |
|  | 2.5. If Y/PY/NI to 2.4: Were these deviations from intended intervention balanced between groups? | | | NA |  |
|  | 2.6 Was an appropriate analysis used to estimate the effect of assignment to intervention? | | | NI |  |
|  | 2.7 If N/PN/NI to 2.6: Was there potential for a substantial impact (on the result) of the failure to analyse participants in the group to which they were randomized? | | | NI |  |
|  | **Risk of bias judgement** | | | **High** |  |
| **Bias due to missing outcome data** | 3.1 Were data for this outcome available for all, or nearly all, participants randomized? | | | N |  |
|  | 3.2 If N/PN/NI to 3.1: Is there evidence that result was not biased by missing outcome data? | | | N |  |
|  | 3.3 If N/PN to 3.2: Could missingness in the outcome depend on its true value? | | | PN |  |
|  | 3.4 If Y/PY/NI to 3.3: Is it likely that missingness in the outcome depended on its true value? | | | NA |  |
|  | **Risk of bias judgement** | | | **Low** |  |
| **Bias in measurement of the outcome** | 4.1 Was the method of measuring the outcome inappropriate? | | | N |  |
|  | 4.2 Could measurement or ascertainment of the outcome have differed between intervention groups? | | | N |  |
|  | 4.3 Were outcome assessors aware of the intervention received by study participants? | | | PY |  |
|  | 4.4 If Y/PY/NI to 4.3: Could assessment of the outcome have been influenced by knowledge of intervention received? | | | PY |  |
|  | 4.5 If Y/PY/NI to 4.4: Is it likely that assessment of the outcome was influenced by knowledge of intervention received? | | | PN |  |
|  | **Risk of bias judgement** | | | **Some concerns** |  |
| **Bias in selection of the reported result** | 5.1 Were the data that produced this result analysed in accordance with a pre-specified analysis plan that was finalized before unblinded outcome data were available for analysis? | | | NI |  |
|  | 5.2 ... multiple eligible outcome measurements (e.g. scales, definitions, time points) within the outcome domain? | | | NI |  |
|  | 5.3 ... multiple eligible analyses of the data? | | | NI |  |
|  | **Risk of bias judgement** | | | **Some concerns** |  |
| **Overall bias** | **Risk of bias judgement** | | | **High** | Controlled parallel group design, not placebo-controlled, not double-blinded. |
|  |  |  |  |  |  |
|  |  |  |  |  |  |
| **Unique ID** | M04STAIT | **Study ID** | Marco04 | **Assessor** |  |
| **Ref or Label** | Marcos 2004 | **Aim** | assignment to intervention (the 'intention-to-treat' effect) |  |  |
| **Experimental** | probiotic | **Comparator** | placebo | **Source** |  |
| **Outcome** | STAI trait | **Results** | -0,03 | **Weight** | 0 |
| **Domain** | **Signalling question** | | | **Response** | **Comments** |
| **Bias arising from the randomization process** | 1.1 Was the allocation sequence random? | | | NI |  |
|  | 1.2 Was the allocation sequence concealed until participants were enrolled and assigned to interventions? | | | NI |  |
|  | 1.3 Did baseline differences between intervention groups suggest a problem with the randomization process? | | | N |  |
|  | **Risk of bias judgement** | | | **Some concerns** |  |
| **Bias due to deviations from intended interventions** | 2.1.Were participants aware of their assigned intervention during the trial? | | | PY |  |
|  | 2.2.Were carers and people delivering the interventions aware of participants' assigned intervention during the trial? | | | PY |  |
|  | 2.3. If Y/PY/NI to 2.1 or 2.2: Were there deviations from the intended intervention that arose because of the experimental context? | | | N |  |
|  | 2.4 If Y/PY to 2.3: Were these deviations likely to have affected the outcome? | | | NA |  |
|  | 2.5. If Y/PY/NI to 2.4: Were these deviations from intended intervention balanced between groups? | | | NA |  |
|  | 2.6 Was an appropriate analysis used to estimate the effect of assignment to intervention? | | | NI |  |
|  | 2.7 If N/PN/NI to 2.6: Was there potential for a substantial impact (on the result) of the failure to analyse participants in the group to which they were randomized? | | | NI |  |
|  | **Risk of bias judgement** | | | **High** |  |
| **Bias due to missing outcome data** | 3.1 Were data for this outcome available for all, or nearly all, participants randomized? | | | N |  |
|  | 3.2 If N/PN/NI to 3.1: Is there evidence that result was not biased by missing outcome data? | | | N |  |
|  | 3.3 If N/PN to 3.2: Could missingness in the outcome depend on its true value? | | | PN |  |
|  | 3.4 If Y/PY/NI to 3.3: Is it likely that missingness in the outcome depended on its true value? | | | NA |  |
|  | **Risk of bias judgement** | | | **Low** |  |
| **Bias in measurement of the outcome** | 4.1 Was the method of measuring the outcome inappropriate? | | | N |  |
|  | 4.2 Could measurement or ascertainment of the outcome have differed between intervention groups? | | | N |  |
|  | 4.3 Were outcome assessors aware of the intervention received by study participants? | | | PY |  |
|  | 4.4 If Y/PY/NI to 4.3: Could assessment of the outcome have been influenced by knowledge of intervention received? | | | PY |  |
|  | 4.5 If Y/PY/NI to 4.4: Is it likely that assessment of the outcome was influenced by knowledge of intervention received? | | | PN |  |
|  | **Risk of bias judgement** | | | **Some concerns** |  |
| **Bias in selection of the reported result** | 5.1 Were the data that produced this result analysed in accordance with a pre-specified analysis plan that was finalized before unblinded outcome data were available for analysis? | | | NI |  |
|  | 5.2 ... multiple eligible outcome measurements (e.g. scales, definitions, time points) within the outcome domain? | | | NI |  |
|  | 5.3 ... multiple eligible analyses of the data? | | | NI |  |
|  | **Risk of bias judgement** | | | **Some concerns** |  |
| **Overall bias** | **Risk of bias judgement** | | | **High** |  |
|  |  |  |  |  |  |
|  |  |  |  |  |  |
| **Unique ID** | S14STAISF | **Study ID** | Sch14 | **Assessor** |  |
| **Ref or Label** | Schmidt | **Aim** | assignment to intervention (the 'intention-to-treat' effect) |  |  |
| **Experimental** | prebiotic FOS | **Comparator** | placebo | **Source** | Non-commercial trial registry record (e.g. ClinicalTrials.gov record) |
| **Outcome** | STAI-state | **Results** | -0,02 | **Weight** | 5.4 |
| **Domain** | **Signalling question** | | | **Response** | **Comments** |
| **Bias arising from the randomization process** | 1.1 Was the allocation sequence random? | | | NI |  |
|  | 1.2 Was the allocation sequence concealed until participants were enrolled and assigned to interventions? | | | NI |  |
|  | 1.3 Did baseline differences between intervention groups suggest a problem with the randomization process? | | | N |  |
|  | **Risk of bias judgement** | | | **Some concerns** |  |
| **Bias due to deviations from intended interventions** | 2.1.Were participants aware of their assigned intervention during the trial? | | | N |  |
|  | 2.2.Were carers and people delivering the interventions aware of participants' assigned intervention during the trial? | | | N |  |
|  | 2.3. If Y/PY/NI to 2.1 or 2.2: Were there deviations from the intended intervention that arose because of the experimental context? | | | NI |  |
|  | 2.4 If Y/PY to 2.3: Were these deviations likely to have affected the outcome? | | | NA |  |
|  | 2.5. If Y/PY/NI to 2.4: Were these deviations from intended intervention balanced between groups? | | | NA |  |
|  | 2.6 Was an appropriate analysis used to estimate the effect of assignment to intervention? | | | N |  |
|  | 2.7 If N/PN/NI to 2.6: Was there potential for a substantial impact (on the result) of the failure to analyse participants in the group to which they were randomized? | | | N |  |
|  | **Risk of bias judgement** | | | **Some concerns** |  |
| **Bias due to missing outcome data** | 3.1 Were data for this outcome available for all, or nearly all, participants randomized? | | | N | Data were available for 93,75% of the participants (to be sufficient it should be 95% based on the Risk of Bias tool assessment RoB2.0) |
|  | 3.2 If N/PN/NI to 3.1: Is there evidence that result was not biased by missing outcome data? | | | N | No sensitivity analysis was performed |
|  | 3.3 If N/PN to 3.2: Could missingness in the outcome depend on its true value? | | | NI | Reasons for drop-out are not reported |
|  | 3.4 If Y/PY/NI to 3.3: Is it likely that missingness in the outcome depended on its true value? | | | PN |  |
|  | **Risk of bias judgement** | | | **Some concerns** |  |
| **Bias in measurement of the outcome** | 4.1 Was the method of measuring the outcome inappropriate? | | | N |  |
|  | 4.2 Could measurement or ascertainment of the outcome have differed between intervention groups? | | | N |  |
|  | 4.3 Were outcome assessors aware of the intervention received by study participants? | | | N |  |
|  | 4.4 If Y/PY/NI to 4.3: Could assessment of the outcome have been influenced by knowledge of intervention received? | | | NA |  |
|  | 4.5 If Y/PY/NI to 4.4: Is it likely that assessment of the outcome was influenced by knowledge of intervention received? | | | NA |  |
|  | **Risk of bias judgement** | | | **Low** |  |
| **Bias in selection of the reported result** | 5.1 Were the data that produced this result analysed in accordance with a pre-specified analysis plan that was finalized before unblinded outcome data were available for analysis? | | | NI |  |
|  | 5.2 ... multiple eligible outcome measurements (e.g. scales, definitions, time points) within the outcome domain? | | | NI | In the trial protocol authors do not mention any self-reported anxiety outcome |
|  | 5.3 ... multiple eligible analyses of the data? | | | NI | SAP are not reported |
|  | **Risk of bias judgement** | | | **Some concerns** |  |
| **Overall bias** | **Risk of bias judgement** | | | **Some concerns** | Overall good quality but some relevant information in regards with the study design are not reported, reason for missing data neither, and a statistical analysis plan is not available |
|  |  |  |  |  |  |
|  |  |  |  |  |  |
| **Unique ID** | S14STAISBG | **Study ID** | Sch14 | **Assessor** |  |
| **Ref or Label** | Schmidt 2014 | **Aim** | assignment to intervention (the 'intention-to-treat' effect) |  |  |
| **Experimental** | prebiotic B-GOS | **Comparator** | placebo | **Source** | Non-commercial trial registry record (e.g. ClinicalTrials.gov record) |
| **Outcome** | STAI-state | **Results** | 0,01 | **Weight** | 5.4 |
| **Domain** | **Signalling question** | | | **Response** | **Comments** |
| **Bias arising from the randomization process** | 1.1 Was the allocation sequence random? | | | NI |  |
|  | 1.2 Was the allocation sequence concealed until participants were enrolled and assigned to interventions? | | | NI |  |
|  | 1.3 Did baseline differences between intervention groups suggest a problem with the randomization process? | | | N |  |
|  | **Risk of bias judgement** | | | **Some concerns** |  |
| **Bias due to deviations from intended interventions** | 2.1.Were participants aware of their assigned intervention during the trial? | | | N |  |
|  | 2.2.Were carers and people delivering the interventions aware of participants' assigned intervention during the trial? | | | N |  |
|  | 2.3. If Y/PY/NI to 2.1 or 2.2: Were there deviations from the intended intervention that arose because of the experimental context? | | | NI |  |
|  | 2.4 If Y/PY to 2.3: Were these deviations likely to have affected the outcome? | | | NA |  |
|  | 2.5. If Y/PY/NI to 2.4: Were these deviations from intended intervention balanced between groups? | | | NA |  |
|  | 2.6 Was an appropriate analysis used to estimate the effect of assignment to intervention? | | | N |  |
|  | 2.7 If N/PN/NI to 2.6: Was there potential for a substantial impact (on the result) of the failure to analyse participants in the group to which they were randomized? | | | N |  |
|  | **Risk of bias judgement** | | | **Some concerns** |  |
| **Bias due to missing outcome data** | 3.1 Were data for this outcome available for all, or nearly all, participants randomized? | | | N |  |
|  | 3.2 If N/PN/NI to 3.1: Is there evidence that result was not biased by missing outcome data? | | | N |  |
|  | 3.3 If N/PN to 3.2: Could missingness in the outcome depend on its true value? | | | NI |  |
|  | 3.4 If Y/PY/NI to 3.3: Is it likely that missingness in the outcome depended on its true value? | | | PN |  |
|  | **Risk of bias judgement** | | | **Some concerns** |  |
| **Bias in measurement of the outcome** | 4.1 Was the method of measuring the outcome inappropriate? | | | N |  |
|  | 4.2 Could measurement or ascertainment of the outcome have differed between intervention groups? | | | N |  |
|  | 4.3 Were outcome assessors aware of the intervention received by study participants? | | | N |  |
|  | 4.4 If Y/PY/NI to 4.3: Could assessment of the outcome have been influenced by knowledge of intervention received? | | | NA |  |
|  | 4.5 If Y/PY/NI to 4.4: Is it likely that assessment of the outcome was influenced by knowledge of intervention received? | | | NA |  |
|  | **Risk of bias judgement** | | | **Low** |  |
| **Bias in selection of the reported result** | 5.1 Were the data that produced this result analysed in accordance with a pre-specified analysis plan that was finalized before unblinded outcome data were available for analysis? | | | NI |  |
|  | 5.2 ... multiple eligible outcome measurements (e.g. scales, definitions, time points) within the outcome domain? | | | NI |  |
|  | 5.3 ... multiple eligible analyses of the data? | | | NI |  |
|  | **Risk of bias judgement** | | | **Some concerns** |  |
| **Overall bias** | **Risk of bias judgement** | | | **Some concerns** |  |
|  |  |  |  |  |  |
|  |  |  |  |  |  |
| **Unique ID** | T19BAIP | **Study ID** | Tra19 | **Assessor** |  |
| **Ref or Label** | Tran 2019 | **Aim** | assignment to intervention (the 'intention-to-treat' effect) |  |  |
| **Experimental** | multispecies probiotic | **Comparator** | placebo | **Source** |  |
| **Outcome** | BAI all conditions | **Results** | 4/ 4.2/ 4.4/ 4.3 | **Weight** | 16.9 |
| **Domain** | **Signalling question** | | | **Response** | **Comments** |
| **Bias arising from the randomization process** | 1.1 Was the allocation sequence random? | | | PY | Randomization performed in Excel. No detailed info about allocation methods, just stated that the leader investigator (not in direct contact with subjects) was aware of the assigned intervention |
|  | 1.2 Was the allocation sequence concealed until participants were enrolled and assigned to interventions? | | | NI |  |
|  | 1.3 Did baseline differences between intervention groups suggest a problem with the randomization process? | | | N |  |
|  | **Risk of bias judgement** | | | **Some concerns** |  |
| **Bias due to deviations from intended interventions** | 2.1.Were participants aware of their assigned intervention during the trial? | | | N |  |
|  | 2.2.Were carers and people delivering the interventions aware of participants' assigned intervention during the trial? | | | N |  |
|  | 2.3. If Y/PY/NI to 2.1 or 2.2: Were there deviations from the intended intervention that arose because of the experimental context? | | | NI |  |
|  | 2.4 If Y/PY to 2.3: Were these deviations likely to have affected the outcome? | | | NA |  |
|  | 2.5. If Y/PY/NI to 2.4: Were these deviations from intended intervention balanced between groups? | | | NA |  |
|  | 2.6 Was an appropriate analysis used to estimate the effect of assignment to intervention? | | | N | Participants who discontinued the intervention were not analysed |
|  | 2.7 If N/PN/NI to 2.6: Was there potential for a substantial impact (on the result) of the failure to analyse participants in the group to which they were randomized? | | | NI |  |
|  | **Risk of bias judgement** | | | **High** |  |
| **Bias due to missing outcome data** | 3.1 Were data for this outcome available for all, or nearly all, participants randomized? | | | N |  |
|  | 3.2 If N/PN/NI to 3.1: Is there evidence that result was not biased by missing outcome data? | | | N |  |
|  | 3.3 If N/PN to 3.2: Could missingness in the outcome depend on its true value? | | | NI |  |
|  | 3.4 If Y/PY/NI to 3.3: Is it likely that missingness in the outcome depended on its true value? | | | PN |  |
|  | **Risk of bias judgement** | | | **Some concerns** |  |
| **Bias in measurement of the outcome** | 4.1 Was the method of measuring the outcome inappropriate? | | | N |  |
|  | 4.2 Could measurement or ascertainment of the outcome have differed between intervention groups? | | | N |  |
|  | 4.3 Were outcome assessors aware of the intervention received by study participants? | | | N |  |
|  | 4.4 If Y/PY/NI to 4.3: Could assessment of the outcome have been influenced by knowledge of intervention received? | | | NA |  |
|  | 4.5 If Y/PY/NI to 4.4: Is it likely that assessment of the outcome was influenced by knowledge of intervention received? | | | NA |  |
|  | **Risk of bias judgement** | | | **Low** |  |
| **Bias in selection of the reported result** | 5.1 Were the data that produced this result analysed in accordance with a pre-specified analysis plan that was finalized before unblinded outcome data were available for analysis? | | | NI |  |
|  | 5.2 ... multiple eligible outcome measurements (e.g. scales, definitions, time points) within the outcome domain? | | | NI |  |
|  | 5.3 ... multiple eligible analyses of the data? | | | NI |  |
|  | **Risk of bias judgement** | | | **Some concerns** |  |
| **Overall bias** | **Risk of bias judgement** | | | **High** | No enough information about the randomization and allocation process, no enough information about adherence to the intervention and reasons of missing data, no applied analysis to account for that, no trial protocol/SAP available. |
|  |  |  |  |  |  |
|  |  |  |  |  |  |
| **Unique ID** | K16STAIS | **Study ID** | Kat16 | **Assessor** |  |
| **Ref or Label** | Kato-Kataoka 2016a | **Aim** | assignment to intervention (the 'intention-to-treat' effect) |  |  |
| **Experimental** | probiotic | **Comparator** | placebo | **Source** | Company-owned trial registry record (e.g. GSK Clinical Study Register record) |
| **Outcome** | STAI-state | **Results** | -0.09 | **Weight** | 7.9 |
| **Domain** | **Signalling question** | | | **Response** | **Comments** |
| **Bias arising from the randomization process** | 1.1 Was the allocation sequence random? | | | PN | Authors never mention any sequence randomization |
|  | 1.2 Was the allocation sequence concealed until participants were enrolled and assigned to interventions? | | | NI |  |
|  | 1.3 Did baseline differences between intervention groups suggest a problem with the randomization process? | | | N |  |
|  | **Risk of bias judgement** | | | **Some concerns** |  |
| **Bias due to deviations from intended interventions** | 2.1.Were participants aware of their assigned intervention during the trial? | | | N |  |
|  | 2.2.Were carers and people delivering the interventions aware of participants' assigned intervention during the trial? | | | N |  |
|  | 2.3. If Y/PY/NI to 2.1 or 2.2: Were there deviations from the intended intervention that arose because of the experimental context? | | | N |  |
|  | 2.4 If Y/PY to 2.3: Were these deviations likely to have affected the outcome? | | | NA |  |
|  | 2.5. If Y/PY/NI to 2.4: Were these deviations from intended intervention balanced between groups? | | | NA |  |
|  | 2.6 Was an appropriate analysis used to estimate the effect of assignment to intervention? | | | Y | Post-allocation exclusion of participants who are no longer eligible independently from the group assignment (students who were not undergoing the final examination) can be considered appropriate |
|  | 2.7 If N/PN/NI to 2.6: Was there potential for a substantial impact (on the result) of the failure to analyse participants in the group to which they were randomized? | | | NA |  |
|  | **Risk of bias judgement** | | | **Low** |  |
| **Bias due to missing outcome data** | 3.1 Were data for this outcome available for all, or nearly all, participants randomized? | | | Y | Participants that dropped out did so before starting the test beverages |
|  | 3.2 If N/PN/NI to 3.1: Is there evidence that result was not biased by missing outcome data? | | | NA |  |
|  | 3.3 If N/PN to 3.2: Could missingness in the outcome depend on its true value? | | | NA |  |
|  | 3.4 If Y/PY/NI to 3.3: Is it likely that missingness in the outcome depended on its true value? | | | NA |  |
|  | **Risk of bias judgement** | | | **Low** |  |
| **Bias in measurement of the outcome** | 4.1 Was the method of measuring the outcome inappropriate? | | | N |  |
|  | 4.2 Could measurement or ascertainment of the outcome have differed between intervention groups? | | | N |  |
|  | 4.3 Were outcome assessors aware of the intervention received by study participants? | | | N |  |
|  | 4.4 If Y/PY/NI to 4.3: Could assessment of the outcome have been influenced by knowledge of intervention received? | | | NA |  |
|  | 4.5 If Y/PY/NI to 4.4: Is it likely that assessment of the outcome was influenced by knowledge of intervention received? | | | NA |  |
|  | **Risk of bias judgement** | | | **Low** |  |
| **Bias in selection of the reported result** | 5.1 Were the data that produced this result analysed in accordance with a pre-specified analysis plan that was finalized before unblinded outcome data were available for analysis? | | | NI | The trial registration record does not mention any of these details |
|  | 5.2 ... multiple eligible outcome measurements (e.g. scales, definitions, time points) within the outcome domain? | | | NI |  |
|  | 5.3 ... multiple eligible analyses of the data? | | | NI |  |
|  | **Risk of bias judgement** | | | **Some concerns** |  |
| **Overall bias** | **Risk of bias judgement** | | | **Some concerns** |  |
|  |  |  |  |  |  |
|  |  |  |  |  |  |
| **Unique ID** | K16bSTAIS | **Study ID** | Kat16b | **Assessor** |  |
| **Ref or Label** | Kato-Kataoka 2016b | **Aim** | assignment to intervention (the 'intention-to-treat' effect) |  |  |
| **Experimental** | probiotic | **Comparator** | placebo | **Source** |  |
| **Outcome** | STAI-state | **Results** | 0.21 | **Weight** | 8 |
| **Domain** | **Signalling question** | | | **Response** | **Comments** |
| **Bias arising from the randomization process** | 1.1 Was the allocation sequence random? | | | N | It was based on the participants background data |
|  | 1.2 Was the allocation sequence concealed until participants were enrolled and assigned to interventions? | | | NI |  |
|  | 1.3 Did baseline differences between intervention groups suggest a problem with the randomization process? | | | Y | STAI scores at baseline significantly differed between probiotic and placebo group (p<0,05) |
|  | **Risk of bias judgement** | | | **High** |  |
| **Bias due to deviations from intended interventions** | 2.1.Were participants aware of their assigned intervention during the trial? | | | N |  |
|  | 2.2.Were carers and people delivering the interventions aware of participants' assigned intervention during the trial? | | | N |  |
|  | 2.3. If Y/PY/NI to 2.1 or 2.2: Were there deviations from the intended intervention that arose because of the experimental context? | | | N |  |
|  | 2.4 If Y/PY to 2.3: Were these deviations likely to have affected the outcome? | | | NA |  |
|  | 2.5. If Y/PY/NI to 2.4: Were these deviations from intended intervention balanced between groups? | | | NA |  |
|  | 2.6 Was an appropriate analysis used to estimate the effect of assignment to intervention? | | | Y | Post-allocation exclusion of participants who are no longer eligible independently from the group assignment (students who were not undergoing the final examination) can be considered appropriate |
|  | 2.7 If N/PN/NI to 2.6: Was there potential for a substantial impact (on the result) of the failure to analyse participants in the group to which they were randomized? | | | NA |  |
|  | **Risk of bias judgement** | | | **Low** |  |
| **Bias due to missing outcome data** | 3.1 Were data for this outcome available for all, or nearly all, participants randomized? | | | N |  |
|  | 3.2 If N/PN/NI to 3.1: Is there evidence that result was not biased by missing outcome data? | | | N |  |
|  | 3.3 If N/PN to 3.2: Could missingness in the outcome depend on its true value? | | | N | Missing data comes from participants excluded due to failure in gaining enough credits to undergo the examination |
|  | 3.4 If Y/PY/NI to 3.3: Is it likely that missingness in the outcome depended on its true value? | | | NA |  |
|  | **Risk of bias judgement** | | | **Low** |  |
| **Bias in measurement of the outcome** | 4.1 Was the method of measuring the outcome inappropriate? | | | N |  |
|  | 4.2 Could measurement or ascertainment of the outcome have differed between intervention groups? | | | N |  |
|  | 4.3 Were outcome assessors aware of the intervention received by study participants? | | | N |  |
|  | 4.4 If Y/PY/NI to 4.3: Could assessment of the outcome have been influenced by knowledge of intervention received? | | | NA |  |
|  | 4.5 If Y/PY/NI to 4.4: Is it likely that assessment of the outcome was influenced by knowledge of intervention received? | | | NA |  |
|  | **Risk of bias judgement** | | | **Low** |  |
| **Bias in selection of the reported result** | 5.1 Were the data that produced this result analysed in accordance with a pre-specified analysis plan that was finalized before unblinded outcome data were available for analysis? | | | NI |  |
|  | 5.2 ... multiple eligible outcome measurements (e.g. scales, definitions, time points) within the outcome domain? | | | NI |  |
|  | 5.3 ... multiple eligible analyses of the data? | | | NI |  |
|  | **Risk of bias judgement** | | | **Some concerns** |  |
| **Overall bias** | **Risk of bias judgement** | | | **High** | There might have been some allocation problems or occurrence of confounding factors between the background data recording and baseline measurement as the STAI scores of the active group is significantly higher in respect with the placebo group at baseline. |

**Appendix 3: Risk of Bias Assessment of stress studies**

| **Unique ID** | A16 | **Study ID** | And16 | **Assessor** |  |
| --- | --- | --- | --- | --- | --- |
| **Ref or Label** | Andersson (2016) | **Aim** | assignment to intervention (the 'intention-to-treat' effect) |  |  |
| **Experimental** | probiotic | **Comparator** | placebo | **Source** |  |
| **Outcome** | salivary cortisol and immunoglobulin A | **Results** |  | **Weight** | 1 |
| **Domain** | **Signalling question** | | | **Response** | **Comments** |
| **Bias arising from the randomization process** | 1.1 Was the allocation sequence random? | | | NI |  |
|  | 1.2 Was the allocation sequence concealed until participants were enrolled and assigned to interventions? | | | NI |  |
|  | 1.3 Did baseline differences between intervention groups suggest a problem with the randomization process? | | | N |  |
|  | **Risk of bias judgement** | | | **Some concerns** |  |
| **Bias due to deviations from intended interventions** | 2.1.Were participants aware of their assigned intervention during the trial? | | | N |  |
|  | 2.2.Were carers and people delivering the interventions aware of participants' assigned intervention during the trial? | | | N |  |
|  | 2.3. If Y/PY/NI to 2.1 or 2.2: Were there deviations from the intended intervention that arose because of the experimental context? | | | NA |  |
|  | 2.4 If Y/PY to 2.3: Were these deviations likely to have affected the outcome? | | | NA |  |
|  | 2.5. If Y/PY/NI to 2.4: Were these deviations from intended intervention balanced between groups? | | | NA |  |
|  | 2.6 Was an appropriate analysis used to estimate the effect of assignment to intervention? | | | NI |  |
|  | 2.7 If N/PN/NI to 2.6: Was there potential for a substantial impact (on the result) of the failure to analyse participants in the group to which they were randomized? | | | N |  |
|  | **Risk of bias judgement** | | | **Some concerns** |  |
| **Bias due to missing outcome data** | 3.1 Were data for this outcome available for all, or nearly all, participants randomized? | | | Y |  |
|  | 3.2 If N/PN/NI to 3.1: Is there evidence that result was not biased by missing outcome data? | | | NA |  |
|  | 3.3 If N/PN to 3.2: Could missingness in the outcome depend on its true value? | | | NA |  |
|  | 3.4 If Y/PY/NI to 3.3: Is it likely that missingness in the outcome depended on its true value? | | | NA |  |
|  | **Risk of bias judgement** | | | **Low** |  |
| **Bias in measurement of the outcome** | 4.1 Was the method of measuring the outcome inappropriate? | | | N |  |
|  | 4.2 Could measurement or ascertainment of the outcome have differed between intervention groups? | | | N |  |
|  | 4.3 Were outcome assessors aware of the intervention received by study participants? | | | N |  |
|  | 4.4 If Y/PY/NI to 4.3: Could assessment of the outcome have been influenced by knowledge of intervention received? | | | NA |  |
|  | 4.5 If Y/PY/NI to 4.4: Is it likely that assessment of the outcome was influenced by knowledge of intervention received? | | | NA |  |
|  | **Risk of bias judgement** | | | **Low** |  |
| **Bias in selection of the reported result** | 5.1 Were the data that produced this result analysed in accordance with a pre-specified analysis plan that was finalized before unblinded outcome data were available for analysis? | | | NI |  |
|  | 5.2 ... multiple eligible outcome measurements (e.g. scales, definitions, time points) within the outcome domain? | | | NI |  |
|  | 5.3 ... multiple eligible analyses of the data? | | | NI |  |
|  | **Risk of bias judgement** | | | **Some concerns** |  |
| **Overall bias** | **Risk of bias judgement** | | | **Some concerns** |  |
|  |  |  |  |  |  |
|  |  |  |  |  |  |
| **Unique ID** | C16 | **Study ID** | Cul16 | **Assessor** |  |
| **Ref or Label** | Culpepper (2016) | **Aim** | assignment to intervention (the 'intention-to-treat' effect) |  |  |
| **Experimental** | probiotic | **Comparator** | placebo | **Source** |  |
| **Outcome** | self-reported stress | **Results** |  | **Weight** | 1 |
| **Domain** | **Signalling question** | | | **Response** | **Comments** |
| **Bias arising from the randomization process** | 1.1 Was the allocation sequence random? | | | Y |  |
|  | 1.2 Was the allocation sequence concealed until participants were enrolled and assigned to interventions? | | | PY |  |
|  | 1.3 Did baseline differences between intervention groups suggest a problem with the randomization process? | | | N |  |
|  | **Risk of bias judgement** | | | **Low** |  |
| **Bias due to deviations from intended interventions** | 2.1.Were participants aware of their assigned intervention during the trial? | | | N |  |
|  | 2.2.Were carers and people delivering the interventions aware of participants' assigned intervention during the trial? | | | N |  |
|  | 2.3. If Y/PY/NI to 2.1 or 2.2: Were there deviations from the intended intervention that arose because of the experimental context? | | | NA |  |
|  | 2.4 If Y/PY to 2.3: Were these deviations likely to have affected the outcome? | | | NA |  |
|  | 2.5. If Y/PY/NI to 2.4: Were these deviations from intended intervention balanced between groups? | | | NA |  |
|  | 2.6 Was an appropriate analysis used to estimate the effect of assignment to intervention? | | | Y |  |
|  | 2.7 If N/PN/NI to 2.6: Was there potential for a substantial impact (on the result) of the failure to analyse participants in the group to which they were randomized? | | | NA |  |
|  | **Risk of bias judgement** | | | **Low** |  |
| **Bias due to missing outcome data** | 3.1 Were data for this outcome available for all, or nearly all, participants randomized? | | | Y |  |
|  | 3.2 If N/PN/NI to 3.1: Is there evidence that result was not biased by missing outcome data? | | | NA |  |
|  | 3.3 If N/PN to 3.2: Could missingness in the outcome depend on its true value? | | | NA |  |
|  | 3.4 If Y/PY/NI to 3.3: Is it likely that missingness in the outcome depended on its true value? | | | NA |  |
|  | **Risk of bias judgement** | | | **Low** |  |
| **Bias in measurement of the outcome** | 4.1 Was the method of measuring the outcome inappropriate? | | | N |  |
|  | 4.2 Could measurement or ascertainment of the outcome have differed between intervention groups? | | | N |  |
|  | 4.3 Were outcome assessors aware of the intervention received by study participants? | | | N |  |
|  | 4.4 If Y/PY/NI to 4.3: Could assessment of the outcome have been influenced by knowledge of intervention received? | | | NA |  |
|  | 4.5 If Y/PY/NI to 4.4: Is it likely that assessment of the outcome was influenced by knowledge of intervention received? | | | NA |  |
|  | **Risk of bias judgement** | | | **Low** |  |
| **Bias in selection of the reported result** | 5.1 Were the data that produced this result analysed in accordance with a pre-specified analysis plan that was finalized before unblinded outcome data were available for analysis? | | | NI |  |
|  | 5.2 ... multiple eligible outcome measurements (e.g. scales, definitions, time points) within the outcome domain? | | | NI |  |
|  | 5.3 ... multiple eligible analyses of the data? | | | NI |  |
|  | **Risk of bias judgement** | | | **Some concerns** |  |
| **Overall bias** | **Risk of bias judgement** | | | **Some concerns** |  |
|  |  |  |  |  |  |
|  |  |  |  |  |  |
| **Unique ID** | H11 | **Study ID** | Hug11 | **Assessor** |  |
| **Ref or Label** | Hughes (2011) | **Aim** | assignment to intervention (the 'intention-to-treat' effect) |  |  |
| **Experimental** | prebiotics | **Comparator** | placebo | **Source** |  |
| **Outcome** | self-reported stress | **Results** |  | **Weight** | 1 |
| **Domain** | **Signalling question** | | | **Response** | **Comments** |
| **Bias arising from the randomization process** | 1.1 Was the allocation sequence random? | | | Y |  |
|  | 1.2 Was the allocation sequence concealed until participants were enrolled and assigned to interventions? | | | Y |  |
|  | 1.3 Did baseline differences between intervention groups suggest a problem with the randomization process? | | | PN |  |
|  | **Risk of bias judgement** | | | **Low** |  |
| **Bias due to deviations from intended interventions** | 2.1.Were participants aware of their assigned intervention during the trial? | | | N |  |
|  | 2.2.Were carers and people delivering the interventions aware of participants' assigned intervention during the trial? | | | N |  |
|  | 2.3. If Y/PY/NI to 2.1 or 2.2: Were there deviations from the intended intervention that arose because of the experimental context? | | | NA |  |
|  | 2.4 If Y/PY to 2.3: Were these deviations likely to have affected the outcome? | | | NA |  |
|  | 2.5. If Y/PY/NI to 2.4: Were these deviations from intended intervention balanced between groups? | | | NA |  |
|  | 2.6 Was an appropriate analysis used to estimate the effect of assignment to intervention? | | | PY |  |
|  | 2.7 If N/PN/NI to 2.6: Was there potential for a substantial impact (on the result) of the failure to analyse participants in the group to which they were randomized? | | | NA |  |
|  | **Risk of bias judgement** | | | **Low** |  |
| **Bias due to missing outcome data** | 3.1 Were data for this outcome available for all, or nearly all, participants randomized? | | | N |  |
|  | 3.2 If N/PN/NI to 3.1: Is there evidence that result was not biased by missing outcome data? | | | N |  |
|  | 3.3 If N/PN to 3.2: Could missingness in the outcome depend on its true value? | | | PN |  |
|  | 3.4 If Y/PY/NI to 3.3: Is it likely that missingness in the outcome depended on its true value? | | | NA |  |
|  | **Risk of bias judgement** | | | **Low** |  |
| **Bias in measurement of the outcome** | 4.1 Was the method of measuring the outcome inappropriate? | | | N |  |
|  | 4.2 Could measurement or ascertainment of the outcome have differed between intervention groups? | | | N |  |
|  | 4.3 Were outcome assessors aware of the intervention received by study participants? | | | N |  |
|  | 4.4 If Y/PY/NI to 4.3: Could assessment of the outcome have been influenced by knowledge of intervention received? | | | NA |  |
|  | 4.5 If Y/PY/NI to 4.4: Is it likely that assessment of the outcome was influenced by knowledge of intervention received? | | | NA |  |
|  | **Risk of bias judgement** | | | **Low** |  |
| **Bias in selection of the reported result** | 5.1 Were the data that produced this result analysed in accordance with a pre-specified analysis plan that was finalized before unblinded outcome data were available for analysis? | | | NI |  |
|  | 5.2 ... multiple eligible outcome measurements (e.g. scales, definitions, time points) within the outcome domain? | | | NI |  |
|  | 5.3 ... multiple eligible analyses of the data? | | | NI |  |
|  | **Risk of bias judgement** | | | **Some concerns** |  |
| **Overall bias** | **Risk of bias judgement** | | | **Some concerns** |  |
|  |  |  |  |  |  |
|  |  |  |  |  |  |
| **Unique ID** | M17 | **Study ID** | Mol17 | **Assessor** |  |
| **Ref or Label** | Moller (2017) | **Aim** | assignment to intervention (the 'intention-to-treat' effect) |  |  |
| **Experimental** | probiotic | **Comparator** | placebo | **Source** |  |
| **Outcome** | PASAT and blood pressure | **Results** |  | **Weight** | 1 |
| **Domain** | **Signalling question** | | | **Response** | **Comments** |
| **Bias arising from the randomization process** | 1.1 Was the allocation sequence random? | | | Y |  |
|  | 1.2 Was the allocation sequence concealed until participants were enrolled and assigned to interventions? | | | PN |  |
|  | 1.3 Did baseline differences between intervention groups suggest a problem with the randomization process? | | | PN |  |
|  | **Risk of bias judgement** | | | **High** |  |
| **Bias due to deviations from intended interventions** | 2.1.Were participants aware of their assigned intervention during the trial? | | | N |  |
|  | 2.2.Were carers and people delivering the interventions aware of participants' assigned intervention during the trial? | | | N |  |
|  | 2.3. If Y/PY/NI to 2.1 or 2.2: Were there deviations from the intended intervention that arose because of the experimental context? | | | NA |  |
|  | 2.4 If Y/PY to 2.3: Were these deviations likely to have affected the outcome? | | | NA |  |
|  | 2.5. If Y/PY/NI to 2.4: Were these deviations from intended intervention balanced between groups? | | | NA |  |
|  | 2.6 Was an appropriate analysis used to estimate the effect of assignment to intervention? | | | PY |  |
|  | 2.7 If N/PN/NI to 2.6: Was there potential for a substantial impact (on the result) of the failure to analyse participants in the group to which they were randomized? | | | NA |  |
|  | **Risk of bias judgement** | | | **Low** |  |
| **Bias due to missing outcome data** | 3.1 Were data for this outcome available for all, or nearly all, participants randomized? | | | N |  |
|  | 3.2 If N/PN/NI to 3.1: Is there evidence that result was not biased by missing outcome data? | | | N |  |
|  | 3.3 If N/PN to 3.2: Could missingness in the outcome depend on its true value? | | | PY | 5 subjects in the intervention group discontinued due to gastro-intestinal side effects, however the number of drop-outs is not sufficiently high to claim that the missingness of the data is likely to depends on its true value |
|  | 3.4 If Y/PY/NI to 3.3: Is it likely that missingness in the outcome depended on its true value? | | | PN |  |
|  | **Risk of bias judgement** | | | **Some concerns** |  |
| **Bias in measurement of the outcome** | 4.1 Was the method of measuring the outcome inappropriate? | | | N |  |
|  | 4.2 Could measurement or ascertainment of the outcome have differed between intervention groups? | | | N |  |
|  | 4.3 Were outcome assessors aware of the intervention received by study participants? | | | PY |  |
|  | 4.4 If Y/PY/NI to 4.3: Could assessment of the outcome have been influenced by knowledge of intervention received? | | | PN |  |
|  | 4.5 If Y/PY/NI to 4.4: Is it likely that assessment of the outcome was influenced by knowledge of intervention received? | | | NA |  |
|  | **Risk of bias judgement** | | | **Low** |  |
| **Bias in selection of the reported result** | 5.1 Were the data that produced this result analysed in accordance with a pre-specified analysis plan that was finalized before unblinded outcome data were available for analysis? | | | NI |  |
|  | 5.2 ... multiple eligible outcome measurements (e.g. scales, definitions, time points) within the outcome domain? | | | NI |  |
|  | 5.3 ... multiple eligible analyses of the data? | | | NI |  |
|  | **Risk of bias judgement** | | | **Some concerns** |  |
| **Overall bias** | **Risk of bias judgement** | | | **High** |  |
|  |  |  |  |  |  |
|  |  |  |  |  |  |
| **Unique ID** | K20 | **Study ID** | Kar20 | **Assessor** |  |
| **Ref or Label** | Karbownik (2020) | **Aim** | assignment to intervention (the 'intention-to-treat' effect) |  |  |
| **Experimental** | probiotic | **Comparator** | placebo | **Source** |  |
| **Outcome** | salivary cortisol, metanephrine and pulse rate | **Results** |  | **Weight** | 1 |
| **Domain** | **Signalling question** | | | **Response** | **Comments** |
| **Bias arising from the randomization process** | 1.1 Was the allocation sequence random? | | | Y |  |
|  | 1.2 Was the allocation sequence concealed until participants were enrolled and assigned to interventions? | | | Y |  |
|  | 1.3 Did baseline differences between intervention groups suggest a problem with the randomization process? | | | N |  |
|  | **Risk of bias judgement** | | | **Low** |  |
| **Bias due to deviations from intended interventions** | 2.1.Were participants aware of their assigned intervention during the trial? | | | N |  |
|  | 2.2.Were carers and people delivering the interventions aware of participants' assigned intervention during the trial? | | | N |  |
|  | 2.3. If Y/PY/NI to 2.1 or 2.2: Were there deviations from the intended intervention that arose because of the experimental context? | | | NA |  |
|  | 2.4 If Y/PY to 2.3: Were these deviations likely to have affected the outcome? | | | NA |  |
|  | 2.5. If Y/PY/NI to 2.4: Were these deviations from intended intervention balanced between groups? | | | NA |  |
|  | 2.6 Was an appropriate analysis used to estimate the effect of assignment to intervention? | | | PY |  |
|  | 2.7 If N/PN/NI to 2.6: Was there potential for a substantial impact (on the result) of the failure to analyse participants in the group to which they were randomized? | | | NA |  |
|  | **Risk of bias judgement** | | | **Low** |  |
| **Bias due to missing outcome data** | 3.1 Were data for this outcome available for all, or nearly all, participants randomized? | | | N |  |
|  | 3.2 If N/PN/NI to 3.1: Is there evidence that result was not biased by missing outcome data? | | | PY | ITT = PP analyses |
|  | 3.3 If N/PN to 3.2: Could missingness in the outcome depend on its true value? | | | NA |  |
|  | 3.4 If Y/PY/NI to 3.3: Is it likely that missingness in the outcome depended on its true value? | | | NA |  |
|  | **Risk of bias judgement** | | | **Low** |  |
| **Bias in measurement of the outcome** | 4.1 Was the method of measuring the outcome inappropriate? | | | N |  |
|  | 4.2 Could measurement or ascertainment of the outcome have differed between intervention groups? | | | N |  |
|  | 4.3 Were outcome assessors aware of the intervention received by study participants? | | | N |  |
|  | 4.4 If Y/PY/NI to 4.3: Could assessment of the outcome have been influenced by knowledge of intervention received? | | | NA |  |
|  | 4.5 If Y/PY/NI to 4.4: Is it likely that assessment of the outcome was influenced by knowledge of intervention received? | | | NA |  |
|  | **Risk of bias judgement** | | | **Low** |  |
| **Bias in selection of the reported result** | 5.1 Were the data that produced this result analysed in accordance with a pre-specified analysis plan that was finalized before unblinded outcome data were available for analysis? | | | NI |  |
|  | 5.2 ... multiple eligible outcome measurements (e.g. scales, definitions, time points) within the outcome domain? | | | NI |  |
|  | 5.3 ... multiple eligible analyses of the data? | | | NI |  |
|  | **Risk of bias judgement** | | | **Some concerns** |  |
| **Overall bias** | **Risk of bias judgement** | | | **Some concerns** |  |
|  |  |  |  |  |  |
|  |  |  |  |  |  |
| **Unique ID** | K09 | **Study ID** | Kit09 | **Assessor** |  |
| **Ref or Label** | Kitaoka (2009) | **Aim** | assignment to intervention (the 'intention-to-treat' effect) |  |  |
| **Experimental** | prebiotic | **Comparator** | placebo | **Source** |  |
| **Outcome** | salivary cortisol and immunoglobulin A, urinary 8-hydroxydeoxyguanosine | **Results** |  | **Weight** | 1 |
| **Domain** | **Signalling question** | | | **Response** | **Comments** |
| **Bias arising from the randomization process** | 1.1 Was the allocation sequence random? | | | NI |  |
|  | 1.2 Was the allocation sequence concealed until participants were enrolled and assigned to interventions? | | | NI |  |
|  | 1.3 Did baseline differences between intervention groups suggest a problem with the randomization process? | | | PN |  |
|  | **Risk of bias judgement** | | | **Some concerns** |  |
| **Bias due to deviations from intended interventions** | 2.1.Were participants aware of their assigned intervention during the trial? | | | N |  |
|  | 2.2.Were carers and people delivering the interventions aware of participants' assigned intervention during the trial? | | | N |  |
|  | 2.3. If Y/PY/NI to 2.1 or 2.2: Were there deviations from the intended intervention that arose because of the experimental context? | | | NA |  |
|  | 2.4 If Y/PY to 2.3: Were these deviations likely to have affected the outcome? | | | NA |  |
|  | 2.5. If Y/PY/NI to 2.4: Were these deviations from intended intervention balanced between groups? | | | NA |  |
|  | 2.6 Was an appropriate analysis used to estimate the effect of assignment to intervention? | | | NI |  |
|  | 2.7 If N/PN/NI to 2.6: Was there potential for a substantial impact (on the result) of the failure to analyse participants in the group to which they were randomized? | | | NI |  |
|  | **Risk of bias judgement** | | | **High** | No flow diagram |
| **Bias due to missing outcome data** | 3.1 Were data for this outcome available for all, or nearly all, participants randomized? | | | NI |  |
|  | 3.2 If N/PN/NI to 3.1: Is there evidence that result was not biased by missing outcome data? | | | PN |  |
|  | 3.3 If N/PN to 3.2: Could missingness in the outcome depend on its true value? | | | NI |  |
|  | 3.4 If Y/PY/NI to 3.3: Is it likely that missingness in the outcome depended on its true value? | | | NI |  |
|  | **Risk of bias judgement** | | | **High** |  |
| **Bias in measurement of the outcome** | 4.1 Was the method of measuring the outcome inappropriate? | | | N |  |
|  | 4.2 Could measurement or ascertainment of the outcome have differed between intervention groups? | | | N |  |
|  | 4.3 Were outcome assessors aware of the intervention received by study participants? | | | N |  |
|  | 4.4 If Y/PY/NI to 4.3: Could assessment of the outcome have been influenced by knowledge of intervention received? | | | NA |  |
|  | 4.5 If Y/PY/NI to 4.4: Is it likely that assessment of the outcome was influenced by knowledge of intervention received? | | | NA |  |
|  | **Risk of bias judgement** | | | **Low** |  |
| **Bias in selection of the reported result** | 5.1 Were the data that produced this result analysed in accordance with a pre-specified analysis plan that was finalized before unblinded outcome data were available for analysis? | | | NI |  |
|  | 5.2 ... multiple eligible outcome measurements (e.g. scales, definitions, time points) within the outcome domain? | | | NI |  |
|  | 5.3 ... multiple eligible analyses of the data? | | | NI |  |
|  | **Risk of bias judgement** | | | **Some concerns** |  |
| **Overall bias** | **Risk of bias judgement** | | | **High** |  |
|  |  |  |  |  |  |
|  |  |  |  |  |  |
| **Unique ID** | M04 | **Study ID** | Marcu04 | **Assessor** |  |
| **Ref or Label** | Marcus (2004) | **Aim** | assignment to intervention (the 'intention-to-treat' effect) |  |  |
| **Experimental** | probiotic | **Comparator** | placebo | **Source** |  |
| **Outcome** | serum cortisol | **Results** |  | **Weight** | 1 |
| **Domain** | **Signalling question** | | | **Response** | **Comments** |
| **Bias arising from the randomization process** | 1.1 Was the allocation sequence random? | | | NI |  |
|  | 1.2 Was the allocation sequence concealed until participants were enrolled and assigned to interventions? | | | NI |  |
|  | 1.3 Did baseline differences between intervention groups suggest a problem with the randomization process? | | | N |  |
|  | **Risk of bias judgement** | | | **Some concerns** |  |
| **Bias due to deviations from intended interventions** | 2.1.Were participants aware of their assigned intervention during the trial? | | | PY | Study not described as blind/double blind |
|  | 2.2.Were carers and people delivering the interventions aware of participants' assigned intervention during the trial? | | | PY |  |
|  | 2.3. If Y/PY/NI to 2.1 or 2.2: Were there deviations from the intended intervention that arose because of the experimental context? | | | N |  |
|  | 2.4 If Y/PY to 2.3: Were these deviations likely to have affected the outcome? | | | NA |  |
|  | 2.5. If Y/PY/NI to 2.4: Were these deviations from intended intervention balanced between groups? | | | NA |  |
|  | 2.6 Was an appropriate analysis used to estimate the effect of assignment to intervention? | | | NI |  |
|  | 2.7 If N/PN/NI to 2.6: Was there potential for a substantial impact (on the result) of the failure to analyse participants in the group to which they were randomized? | | | NI |  |
|  | **Risk of bias judgement** | | | **High** |  |
| **Bias due to missing outcome data** | 3.1 Were data for this outcome available for all, or nearly all, participants randomized? | | | N |  |
|  | 3.2 If N/PN/NI to 3.1: Is there evidence that result was not biased by missing outcome data? | | | N |  |
|  | 3.3 If N/PN to 3.2: Could missingness in the outcome depend on its true value? | | | PN |  |
|  | 3.4 If Y/PY/NI to 3.3: Is it likely that missingness in the outcome depended on its true value? | | | NA |  |
|  | **Risk of bias judgement** | | | **Low** |  |
| **Bias in measurement of the outcome** | 4.1 Was the method of measuring the outcome inappropriate? | | | N |  |
|  | 4.2 Could measurement or ascertainment of the outcome have differed between intervention groups? | | | N |  |
|  | 4.3 Were outcome assessors aware of the intervention received by study participants? | | | PY |  |
|  | 4.4 If Y/PY/NI to 4.3: Could assessment of the outcome have been influenced by knowledge of intervention received? | | | PY |  |
|  | 4.5 If Y/PY/NI to 4.4: Is it likely that assessment of the outcome was influenced by knowledge of intervention received? | | | PN |  |
|  | **Risk of bias judgement** | | | **Some concerns** |  |
| **Bias in selection of the reported result** | 5.1 Were the data that produced this result analysed in accordance with a pre-specified analysis plan that was finalized before unblinded outcome data were available for analysis? | | | NI |  |
|  | 5.2 ... multiple eligible outcome measurements (e.g. scales, definitions, time points) within the outcome domain? | | | NI |  |
|  | 5.3 ... multiple eligible analyses of the data? | | | NI |  |
|  | **Risk of bias judgement** | | | **Some concerns** |  |
| **Overall bias** | **Risk of bias judgement** | | | **High** |  |
|  |  |  |  |  |  |
|  |  |  |  |  |  |
| **Unique ID** | S14 | **Study ID** | Sch14 | **Assessor** |  |
| **Ref or Label** | Schmidt (2014) | **Aim** | assignment to intervention (the 'intention-to-treat' effect) |  |  |
| **Experimental** | prebiotic | **Comparator** | placebo | **Source** | Non-commercial trial registry record (e.g. ClinicalTrials.gov record) |
| **Outcome** | PSS-10, salivary cortisol | **Results** |  | **Weight** | 1 |
| **Domain** | **Signalling question** | | | **Response** | **Comments** |
| **Bias arising from the randomization process** | 1.1 Was the allocation sequence random? | | | NI |  |
|  | 1.2 Was the allocation sequence concealed until participants were enrolled and assigned to interventions? | | | NI |  |
|  | 1.3 Did baseline differences between intervention groups suggest a problem with the randomization process? | | | N |  |
|  | **Risk of bias judgement** | | | **Some concerns** |  |
| **Bias due to deviations from intended interventions** | 2.1.Were participants aware of their assigned intervention during the trial? | | | N |  |
|  | 2.2.Were carers and people delivering the interventions aware of participants' assigned intervention during the trial? | | | N |  |
|  | 2.3. If Y/PY/NI to 2.1 or 2.2: Were there deviations from the intended intervention that arose because of the experimental context? | | | NA |  |
|  | 2.4 If Y/PY to 2.3: Were these deviations likely to have affected the outcome? | | | NA |  |
|  | 2.5. If Y/PY/NI to 2.4: Were these deviations from intended intervention balanced between groups? | | | NA |  |
|  | 2.6 Was an appropriate analysis used to estimate the effect of assignment to intervention? | | | N |  |
|  | 2.7 If N/PN/NI to 2.6: Was there potential for a substantial impact (on the result) of the failure to analyse participants in the group to which they were randomized? | | | N |  |
|  | **Risk of bias judgement** | | | **Some concerns** |  |
| **Bias due to missing outcome data** | 3.1 Were data for this outcome available for all, or nearly all, participants randomized? | | | N |  |
|  | 3.2 If N/PN/NI to 3.1: Is there evidence that result was not biased by missing outcome data? | | | N |  |
|  | 3.3 If N/PN to 3.2: Could missingness in the outcome depend on its true value? | | | NI |  |
|  | 3.4 If Y/PY/NI to 3.3: Is it likely that missingness in the outcome depended on its true value? | | | PN |  |
|  | **Risk of bias judgement** | | | **Some concerns** |  |
| **Bias in measurement of the outcome** | 4.1 Was the method of measuring the outcome inappropriate? | | | N |  |
|  | 4.2 Could measurement or ascertainment of the outcome have differed between intervention groups? | | | N |  |
|  | 4.3 Were outcome assessors aware of the intervention received by study participants? | | | N |  |
|  | 4.4 If Y/PY/NI to 4.3: Could assessment of the outcome have been influenced by knowledge of intervention received? | | | NA |  |
|  | 4.5 If Y/PY/NI to 4.4: Is it likely that assessment of the outcome was influenced by knowledge of intervention received? | | | NA |  |
|  | **Risk of bias judgement** | | | **Low** |  |
| **Bias in selection of the reported result** | 5.1 Were the data that produced this result analysed in accordance with a pre-specified analysis plan that was finalized before unblinded outcome data were available for analysis? | | | NI |  |
|  | 5.2 ... multiple eligible outcome measurements (e.g. scales, definitions, time points) within the outcome domain? | | | NI |  |
|  | 5.3 ... multiple eligible analyses of the data? | | | NI |  |
|  | **Risk of bias judgement** | | | **Some concerns** |  |
| **Overall bias** | **Risk of bias judgement** | | |  |  |
|  |  |  |  |  |  |
|  |  |  |  |  |  |
| **Unique ID** | K16 | **Study ID** | Kat16 | **Assessor** |  |
| **Ref or Label** | Kato-Kataoka (2016) | **Aim** | assignment to intervention (the 'intention-to-treat' effect) |  |  |
| **Experimental** | probiotic | **Comparator** | placebo | **Source** | Company-owned trial registry record (e.g. GSK Clinical Study Register record) |
| **Outcome** | salivary cortisol and alpha amylase, visual analogue stress scale | **Results** |  | **Weight** | 1 |
| **Domain** | **Signalling question** | | | **Response** | **Comments** |
| **Bias arising from the randomization process** | 1.1 Was the allocation sequence random? | | | PN |  |
|  | 1.2 Was the allocation sequence concealed until participants were enrolled and assigned to interventions? | | | NI |  |
|  | 1.3 Did baseline differences between intervention groups suggest a problem with the randomization process? | | | N |  |
|  | **Risk of bias judgement** | | | **Some concerns** |  |
| **Bias due to deviations from intended interventions** | 2.1.Were participants aware of their assigned intervention during the trial? | | | N |  |
|  | 2.2.Were carers and people delivering the interventions aware of participants' assigned intervention during the trial? | | | N |  |
|  | 2.3. If Y/PY/NI to 2.1 or 2.2: Were there deviations from the intended intervention that arose because of the experimental context? | | | NA |  |
|  | 2.4 If Y/PY to 2.3: Were these deviations likely to have affected the outcome? | | | NA |  |
|  | 2.5. If Y/PY/NI to 2.4: Were these deviations from intended intervention balanced between groups? | | | NA |  |
|  | 2.6 Was an appropriate analysis used to estimate the effect of assignment to intervention? | | | Y |  |
|  | 2.7 If N/PN/NI to 2.6: Was there potential for a substantial impact (on the result) of the failure to analyse participants in the group to which they were randomized? | | | NA |  |
|  | **Risk of bias judgement** | | | **Low** |  |
| **Bias due to missing outcome data** | 3.1 Were data for this outcome available for all, or nearly all, participants randomized? | | | Y |  |
|  | 3.2 If N/PN/NI to 3.1: Is there evidence that result was not biased by missing outcome data? | | | NA |  |
|  | 3.3 If N/PN to 3.2: Could missingness in the outcome depend on its true value? | | | NA |  |
|  | 3.4 If Y/PY/NI to 3.3: Is it likely that missingness in the outcome depended on its true value? | | | NA |  |
|  | **Risk of bias judgement** | | | **Low** |  |
| **Bias in measurement of the outcome** | 4.1 Was the method of measuring the outcome inappropriate? | | | N |  |
|  | 4.2 Could measurement or ascertainment of the outcome have differed between intervention groups? | | | N |  |
|  | 4.3 Were outcome assessors aware of the intervention received by study participants? | | | N |  |
|  | 4.4 If Y/PY/NI to 4.3: Could assessment of the outcome have been influenced by knowledge of intervention received? | | | NA |  |
|  | 4.5 If Y/PY/NI to 4.4: Is it likely that assessment of the outcome was influenced by knowledge of intervention received? | | | NA |  |
|  | **Risk of bias judgement** | | | **Low** |  |
| **Bias in selection of the reported result** | 5.1 Were the data that produced this result analysed in accordance with a pre-specified analysis plan that was finalized before unblinded outcome data were available for analysis? | | | NI |  |
|  | 5.2 ... multiple eligible outcome measurements (e.g. scales, definitions, time points) within the outcome domain? | | | NI |  |
|  | 5.3 ... multiple eligible analyses of the data? | | | NI |  |
|  | **Risk of bias judgement** | | | **Some concerns** |  |
| **Overall bias** | **Risk of bias judgement** | | | **Some concerns** |  |
|  |  |  |  |  |  |
|  |  |  |  |  |  |
| **Unique ID** | K16b | **Study ID** | Kat16b | **Assessor** |  |
| **Ref or Label** | Kato-Kataoka (2016) | **Aim** | assignment to intervention (the 'intention-to-treat' effect) |  |  |
| **Experimental** | probiotic | **Comparator** | placebo | **Source** |  |
| **Outcome** | salivary cortisol and immunoglobulin A | **Results** |  | **Weight** | 1 |
| **Domain** | **Signalling question** | | | **Response** | **Comments** |
| **Bias arising from the randomization process** | 1.1 Was the allocation sequence random? | | | N | Based on background data |
|  | 1.2 Was the allocation sequence concealed until participants were enrolled and assigned to interventions? | | | NI |  |
|  | 1.3 Did baseline differences between intervention groups suggest a problem with the randomization process? | | | N |  |
|  | **Risk of bias judgement** | | | **Some concerns** |  |
| **Bias due to deviations from intended interventions** | 2.1.Were participants aware of their assigned intervention during the trial? | | | N |  |
|  | 2.2.Were carers and people delivering the interventions aware of participants' assigned intervention during the trial? | | | N |  |
|  | 2.3. If Y/PY/NI to 2.1 or 2.2: Were there deviations from the intended intervention that arose because of the experimental context? | | | NA |  |
|  | 2.4 If Y/PY to 2.3: Were these deviations likely to have affected the outcome? | | | NA |  |
|  | 2.5. If Y/PY/NI to 2.4: Were these deviations from intended intervention balanced between groups? | | | NA |  |
|  | 2.6 Was an appropriate analysis used to estimate the effect of assignment to intervention? | | | Y |  |
|  | 2.7 If N/PN/NI to 2.6: Was there potential for a substantial impact (on the result) of the failure to analyse participants in the group to which they were randomized? | | | NA |  |
|  | **Risk of bias judgement** | | | **Low** |  |
| **Bias due to missing outcome data** | 3.1 Were data for this outcome available for all, or nearly all, participants randomized? | | | N |  |
|  | 3.2 If N/PN/NI to 3.1: Is there evidence that result was not biased by missing outcome data? | | | N |  |
|  | 3.3 If N/PN to 3.2: Could missingness in the outcome depend on its true value? | | | N |  |
|  | 3.4 If Y/PY/NI to 3.3: Is it likely that missingness in the outcome depended on its true value? | | | NA |  |
|  | **Risk of bias judgement** | | | **Low** |  |
| **Bias in measurement of the outcome** | 4.1 Was the method of measuring the outcome inappropriate? | | | N |  |
|  | 4.2 Could measurement or ascertainment of the outcome have differed between intervention groups? | | | N |  |
|  | 4.3 Were outcome assessors aware of the intervention received by study participants? | | | N |  |
|  | 4.4 If Y/PY/NI to 4.3: Could assessment of the outcome have been influenced by knowledge of intervention received? | | | NA |  |
|  | 4.5 If Y/PY/NI to 4.4: Is it likely that assessment of the outcome was influenced by knowledge of intervention received? | | | NA |  |
|  | **Risk of bias judgement** | | | **Low** |  |
| **Bias in selection of the reported result** | 5.1 Were the data that produced this result analysed in accordance with a pre-specified analysis plan that was finalized before unblinded outcome data were available for analysis? | | | NI |  |
|  | 5.2 ... multiple eligible outcome measurements (e.g. scales, definitions, time points) within the outcome domain? | | | NI |  |
|  | 5.3 ... multiple eligible analyses of the data? | | | NI |  |
|  | **Risk of bias judgement** | | | **Some concerns** |  |
| **Overall bias** | **Risk of bias judgement** | | | **Some concerns** |  |

**Appendix 4**


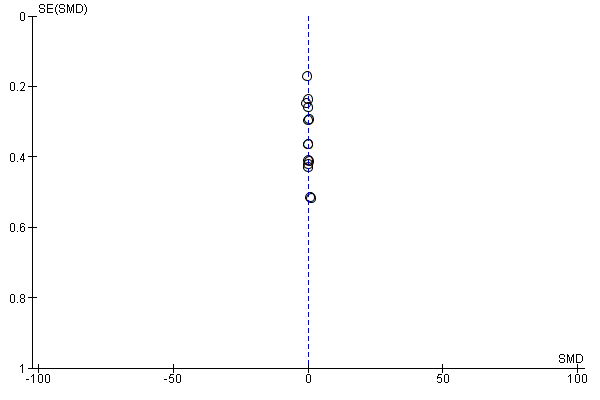


**Funnel plot:** (i) x axis: SMD per each included outcome. (ii) y axis: standard error of the effect estimated.

Larger studies with higher power are placed to the top, lower powered studies towards the bottom instead.

As most studies did not find any significant effect, they all fall on the vertical line. Although there is no sign of asymmetry , given the small amount of included studies and the absence of consistent trends in the effect, we cannot confidently state whether any publication bias might be present. This, together with the absence of pre-specified protocol for most included studies (which arises some concerns in terms of selective reporting within studies) and the exclusion of any unpublished research, warrant caution in the interpretation of the finding of this systematic review.

**Appendix 5**: **PRISMA checklist**

| **Section/topic** | **#** | **Checklist item** | **Reported on page #** |
| --- | --- | --- | --- |
| **TITLE** | | |  |
| Title | 1 | Identify the report as a systematic review, meta-analysis, or both. | 1 |
| **ABSTRACT** | | |  |
| Structured summary | 2 | Provide a structured summary including, as applicable: background; objectives; data sources; study eligibility criteria, participants, and interventions; study appraisal and synthesis methods; results; limitations; conclusions and implications of key findings; systematic review registration number. | 2 |
| **INTRODUCTION** | | |  |
| Rationale | 3 | Describe the rationale for the review in the context of what is already known. | 3 - 5 |
| Objectives | 4 | Provide an explicit statement of questions being addressed with reference to participants, interventions, comparisons, outcomes, and study design (PICOS). | 6 |
| **METHODS** | | |  |
| Protocol and registration | 5 | Indicate if a review protocol exists, if and where it can be accessed (e.g., Web address), and, if available, provide registration information including registration number. | 6 |
| Eligibility criteria | 6 | Specify study characteristics (e.g., PICOS, length of follow-up) and report characteristics (e.g., years considered, language, publication status) used as criteria for eligibility, giving rationale. | 6, 32 |
| Information sources | 7 | Describe all information sources (e.g., databases with dates of coverage, contact with study authors to identify additional studies) in the search and date last searched. | 6 |
| Search | 8 | Present full electronic search strategy for at least one database, including any limits used, such that it could be repeated. | 32-35 |
| Study selection | 9 | State the process for selecting studies (i.e., screening, eligibility, included in systematic review, and, if applicable, included in the meta-analysis). | 6 |
| Data collection process | 10 | Describe method of data extraction from reports (e.g., piloted forms, independently, in duplicate) and any processes for obtaining and confirming data from investigators. | 7 |
| Data items | 11 | List and define all variables for which data were sought (e.g., PICOS, funding sources) and any assumptions and simplifications made. | 7 |
| Risk of bias in individual studies | 12 | Describe methods used for assessing risk of bias of individual studies (including specification of whether this was done at the study or outcome level), and how this information is to be used in any data synthesis. | 7 |
| Summary measures | 13 | State the principal summary measures (e.g., risk ratio, difference in means). | 7 |
| Synthesis of results | 14 | Describe the methods of handling data and combining results of studies, if done, including measures of consistency (e.g., I^2^) for each meta-analysis. | 7 |

| **Section/topic** | **#** | **Checklist item** | **Reported on page #** |
| --- | --- | --- | --- |
| Risk of bias across studies | 15 | Specify any assessment of risk of bias that may affect the cumulative evidence (e.g., publication bias, selective reporting within studies). | 7 |
| Additional analyses | 16 | Describe methods of additional analyses (e.g., sensitivity or subgroup analyses, meta-regression), if done, indicating which were pre-specified. | 7 |
| **RESULTS** | | |  |
| Study selection | 17 | Give numbers of studies screened, assessed for eligibility, and included in the review, with reasons for exclusions at each stage, ideally with a flow diagram. | 8 |
| Study characteristics | 18 | For each study, present characteristics for which data were extracted (e.g., study size, PICOS, follow-up period) and provide the citations. | 26-28 |
| Risk of bias within studies | 19 | Present data on risk of bias of each study and, if available, any outcome level assessment (see item 12). | 12, 36-77 |
| Results of individual studies | 20 | For all outcomes considered (benefits or harms), present, for each study: (a) simple summary data for each intervention group (b) effect estimates and confidence intervals, ideally with a forest plot. | 26-28, 12 |
| Synthesis of results | 21 | Present results of each meta-analysis done, including confidence intervals and measures of consistency. | 12 |
| Risk of bias across studies | 22 | Present results of any assessment of risk of bias across studies (see Item 15). | 36-77 |
| Additional analysis | 23 | Give results of additional analyses, if done (e.g., sensitivity or subgroup analyses, meta-regression [see Item 16]). | 12 |
| **DISCUSSION** | | |  |
| Summary of evidence | 24 | Summarize the main findings including the strength of evidence for each main outcome; consider their relevance to key groups (e.g., healthcare providers, users, and policy makers). | 13 |
| Limitations | 25 | Discuss limitations at study and outcome level (e.g., risk of bias), and at review-level (e.g., incomplete retrieval of identified research, reporting bias). | 13 |
| Conclusions | 26 | Provide a general interpretation of the results in the context of other evidence, and implications for future research. | 18 |
| **FUNDING** | | |  |
| Funding | 27 | Describe sources of funding for the systematic review and other support (e.g., supply of data); role of funders for the systematic review. | 18 |
